# Genomic adaptations of *Campylobacter jejuni* to long-term human colonization

**DOI:** 10.1186/s13099-021-00469-7

**Published:** 2021-12-10

**Authors:** Samuel J. Bloomfield, Anne C. Midwinter, Patrick J. Biggs, Nigel P. French, Jonathan C. Marshall, David T. S. Hayman, Philip E. Carter, Alison E. Mather, Ahmed Fayaz, Craig Thornley, David J. Kelly, Jackie Benschop

**Affiliations:** 1 Quadram Institute Bioscience, Norwich Research Park, Norwich, Norfolk UK; 2 mEpiLab, Hopkirk Research Institute, Massey University, Palmerston North, 4410 New Zealand; 3 Infectious Disease Research Centre, Hopkirk Research Institute, Massey University, Palmerston North, 4410 New Zealand; 4 School of Fundamental Science, Massey University, Palmerston North, 4410 New Zealand; 5 New Zealand Food Safety Science and Research Centre, Hopkirk Research Institute, Massey University, Palmerston North, 4410 New Zealand; 6 Centre of Research Excellence for Complex Systems, Te Pūnaha Matatini, Auckland, New Zealand; 7 Institute of Environmental Science of Research, 34 Kenepuru Drive, Kenepuru, Porirua, 5022 New Zealand; 8 University of East Anglia, Norwich, Norfolk UK; 9 Regional Public Health, Hutt Hospital, Lower Hutt, 5040 New Zealand; 10 School of Biosciences, The University of Sheffield, Sheffield, South Yorkshire UK

**Keywords:** *Campylobacter*, Genomics, Host adaptation, Phylogenetics

## Abstract

**Background:**

*Campylobacter* is a genus of bacteria that has been isolated from the gastrointestinal tract of humans and animals, and the environments they inhabit around the world. *Campylobacter* adapt to new environments by changes in their gene content and expression, but little is known about how they adapt to long-term human colonization. In this study, the genomes of 31 isolates from a New Zealand patient and 22 isolates from a United Kingdom patient belonging to *Campylobacter jejuni* sequence type 45 (ST45) were compared with 209 ST45 genomes from other sources to identify the mechanisms by which *Campylobacter* adapts to long-term human colonization. In addition, the New Zealand patient had their microbiota investigated using 16S rRNA metabarcoding, and their level of inflammation and immunosuppression analyzed using biochemical tests, to determine how *Campylobacter* adapts to a changing gastrointestinal tract.

**Results:**

There was some evidence that long-term colonization led to genome degradation, but more evidence that *Campylobacter* adapted through the accumulation of non-synonymous single nucleotide polymorphisms (SNPs) and frameshifts in genes involved in cell motility, signal transduction and the major outer membrane protein (MOMP). The New Zealand patient also displayed considerable variation in their microbiome, inflammation and immunosuppression over five months, and the *Campylobacter* collected from this patient could be divided into two subpopulations, the proportion of which correlated with the amount of gastrointestinal inflammation.

**Conclusions:**

This study demonstrates how genomics, phylogenetics, 16S rRNA metabarcoding and biochemical markers can provide insight into how *Campylobacter* adapts to changing environments within human hosts. This study also demonstrates that long-term human colonization selects for changes in *Campylobacter* genes involved in cell motility, signal transduction and the MOMP; and that genetically distinct subpopulations of *Campylobacter* evolve to adapt to the changing gastrointestinal environment.

**Supplementary Information:**

The online version contains supplementary material available at 10.1186/s13099-021-00469-7.

## Background

*Campylobacter* is a genus of bacteria that has been isolated from the gastrointestinal tract of humans and other animals around the world [1], and from the environments these animals inhabit [2]. Some *Campylobacter* species are host generalists, colonizing a wide range of host species [3], others are host-specific [4], and some are pathogenic to the animals they colonize [5]. Population genomics and animal studies have demonstrated that *Campylobacter* adapts to hosts by changing its gene content and expression [6, 7]. As long-term *Campylobacter* colonization in humans is rare, few studies have investigated *Campylobacter* adaption to human hosts [8, 9].

Previously, a patient in New Zealand was identified that had been excreting the same strain of *Campylobacter, C. jejuni* sequence type (ST) 45, for over a decade [8]. The patient had been diagnosed with common variable immune deficiency (CVID) and had suffered from daily diarrhea that varied in severity. Previous genomic analysis and antimicrobial susceptibility testing of *Campylobacter* isolates collected from the patient determined that the patient was consistently colonized with *Campylobacter* over this time period, and that the *Campylobacter* had developed resistance to the antimicrobial agents the patient was prescribed. However, it was unclear to what extent the *Campylobacter* was contributing to the patient’s diarrhea. In this study we compared the genomes of ST45 isolates collected from the patient to those collected from other sources, including another patient from the United Kingdom with long-term colonization, to investigate how *Campylobacter* had adapted to human hosts, and used 16S rRNA metabarcoding and biochemical markers to investigate changes in microbial communities and biochemical markers over the period of long-term *Campylobacter* colonization.

## Results

### Sample and isolate collection

The New Zealand patient submitted three fecal and three serum samples over five months. Three *Campylobacter* isolates were collected from the first fecal sample, and six were collected from the second and third samples. These 15 isolates were combined with the 16 previously sequenced from this patient. An additional 231 *C. jejuni* ST45 isolates were downloaded and passed the quality control thresholds, including 22 from a long-term patient from the United Kingdom (Additional file 1).

### ST45 pan-genome analysis

The 262 ST45 genomes contained a pan-genome of 3041 genes and a core-genome of 1459 genes. The pan-genome also contained 603 pseudogenes, 206 of which the pseudogene or original gene were found in more than 95% of isolates. PlasmidFinder identified a plasmid replicon (IncFII) in one of the ST45 isolates, and RFPlasmid found evidence of plasmid-associated contigs in 109 contigs from 50 ST45 isolates. Amongst the plasmid-associated contigs, three contained antimicrobial resistance (AMR) genes, four contained virulence genes, fourteen were conjugative, six were mobile and 89 were non-mobile. None of the isolates collected from the New Zealand patient contained plasmid-associated contigs, but two of the isolates from the United Kingdom patients did. However, they were non-motile and contained no AMR or virulence genes.

Gene frequency analysis did not find any evidence of specific genes or pseudogenes associated with the 53 long-term *Campylobacter* patient isolates when compared to the 209 other ST45 isolates or the subset of 145 other human ST45 isolates investigated, and linear regression analysis found that the number of genes was not significantly associated with source (p = 0.2216) or country (p = 0.9765). The number of pseudogenes was also not significantly associated with source (p = 0.101), but was significantly associated with country (p = 0.0114), with isolates from the United States containing more pseudogenes. This difference was small and likely due to sampling (Additional file 2).

All isolates collected from the long-term *Campylobacter* patients contained the *bla*_*OXA-61*_ beta-lactamase gene [10] and the T86I mutation in the *gyrA* gene associated with fluoroquinolone resistance [11], as did 152 and two of the 209 other ST45 isolates, respectively. Frameshift and SNP analysis demonstrated that the T86I mutation was the only mutation associated with long-term *Campylobacter* patient isolates, when compared to all the other ST45 isolates or just the other human ST45 isolated investigated. All isolates collected from the New Zealand patient and two non-patient ST45 isolates contained the A2074T mutation in the 23S rRNA gene associated with macrolide resistance [12]. One of the isolates collected from the United Kingdom patient contained the A2075G mutation in the 23S rRNA gene. No long-term patient-specific virulence genes were identified.

**Fig. 1:**
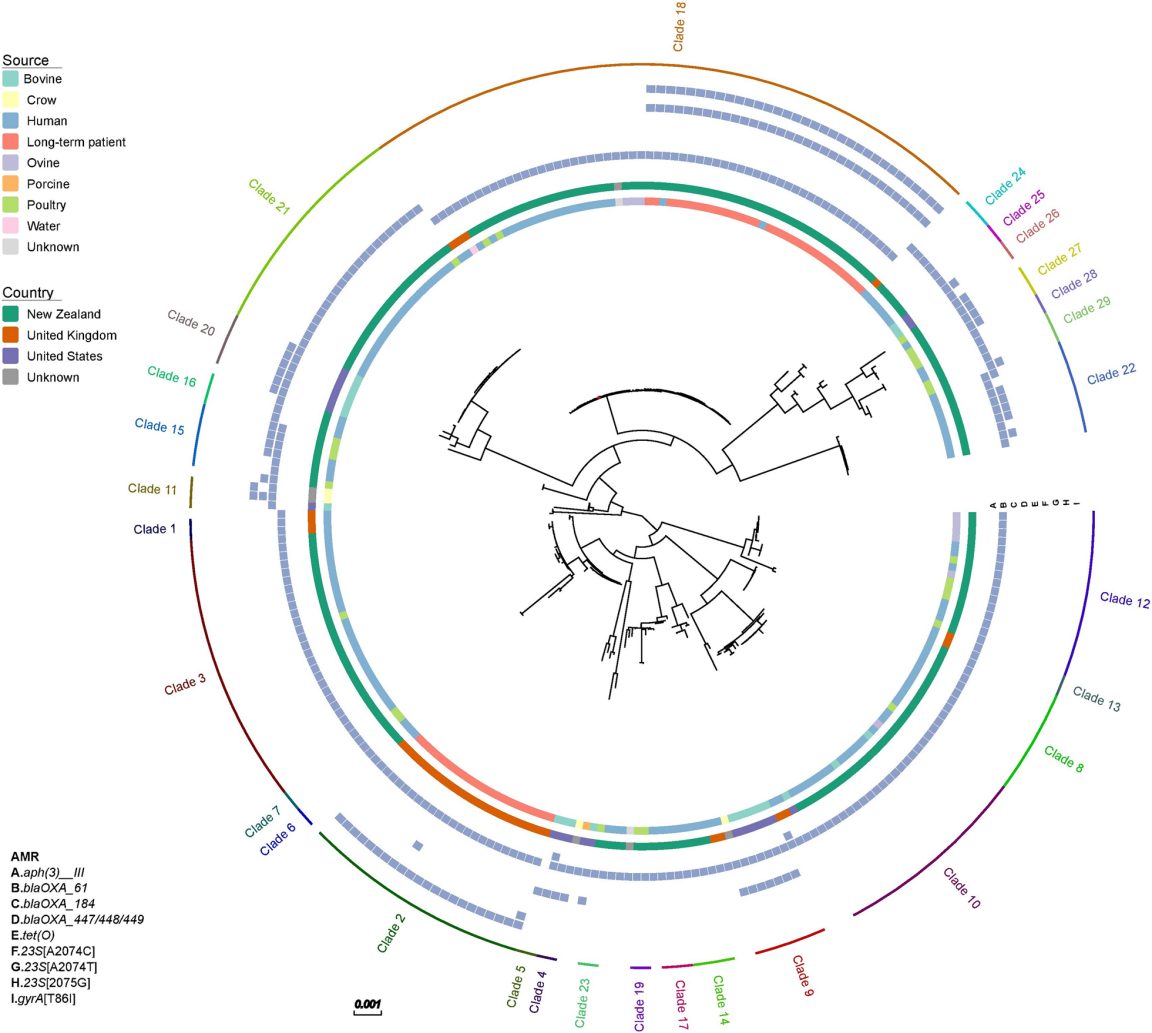
Maximum likelihood tree of 262 ST45 isolates. Colored bars represent isolate metadata and the presence-absence matrix represents antimicrobial resistance genes

Phylogenetic analysis of the ST45 isolates from different sources and countries demonstrated that the sequence type is very diverse (Fig. 1). Clade analysis identified 29 clades amongst the 262 ST45 isolates. Thirteen of these clades consisted of isolates from multiple sources, whilst the remaining clades consisted of isolates from the same source. However, only three of these clades contained more than one non-human source. The isolates from the New Zealand patient were in the same clade as isolates from water, ovine, poultry and other human isolates (Clade 18), whilst no other isolates were found in the same clade as the United Kingdom long-term patient (Clade 2) (Additional file 3). A closer examination of clade 18 revealed that the isolates collected from the New Zealand patient shared 2–123 SNPs with each other, and 22–239 SNPs with other isolates in this clade. They were most closely related to two genomes from New Zealand human sources. These two genomes were the only other ST45 isolates that contained the A2074T mutation in the 23S rRNA gene.

### New Zealand patient phylogenetics

SNP analysis identified 248 non-recombinant SNPs amongst the 31 isolates collected from the New Zealand patient. TempEst found tip dates and distance were correlated (R^2^ = 0.92) (Additional file 4). Phylogenetic modeling estimated that these isolates contained a median substitution rate of 3.69 × 10^–6^ substitutions site^−1^ year^−1^ (95% HPD interval: 2.87–4.49 × 10^–6^ substitutions site^−1^ year^−1^) and shared a common ancestor on 09/05/2004 (median, 95% HPD interval: 11/12/2002–16/07/2005). The sampled ancestor model did not identify isolates that represented ancestors of other isolates collected from the patient.

**Fig. 2:**
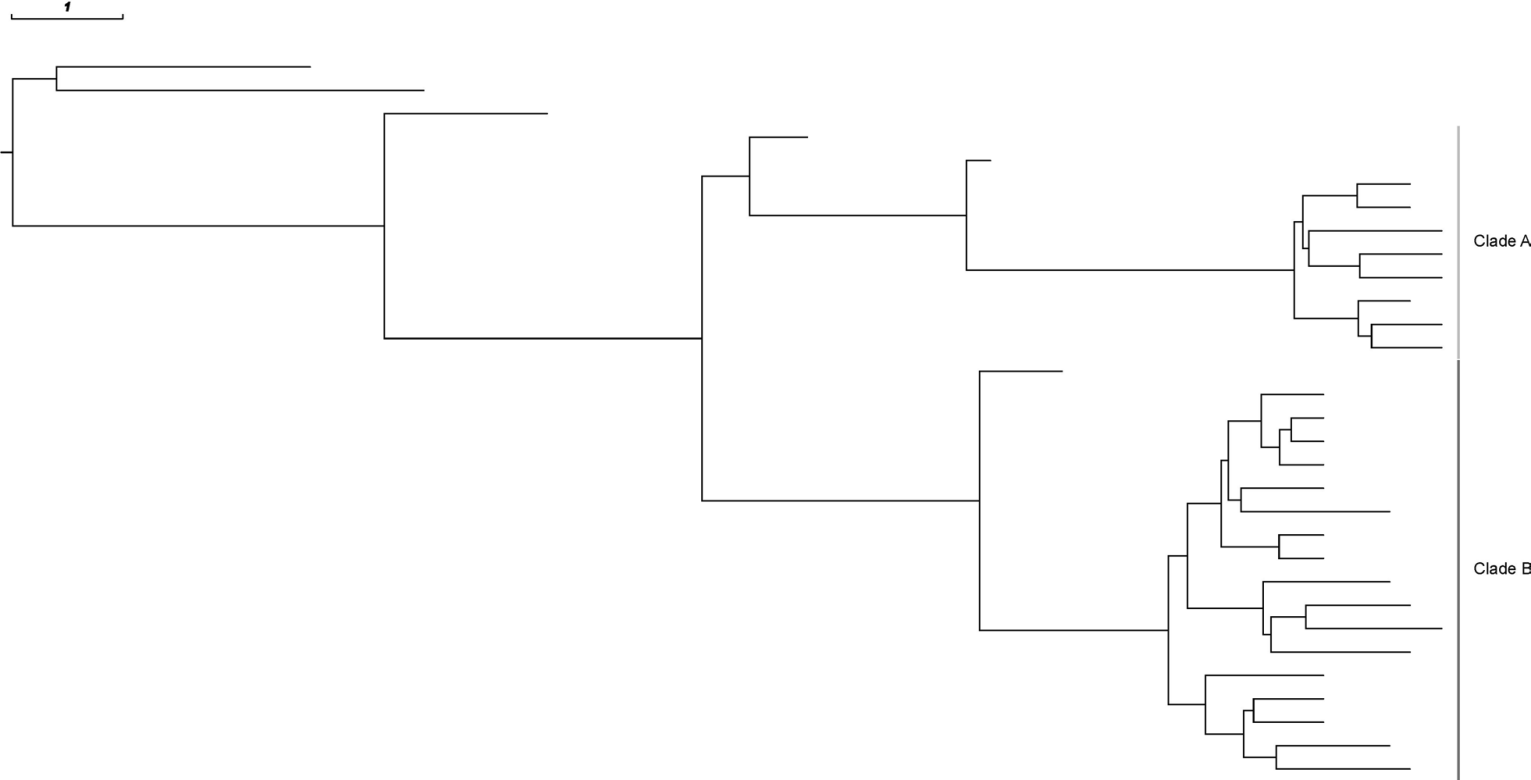
Maximum clade credibility tree of 31 ST45 isolates collected from the same New Zealand patient

Phylogenetic analysis revealed that two clades had evolved within the New Zealand patient representing separate subpopulations (Fig. 2). These clades shared a common ancestor on 08/07/2010 (median, 95% HPD interval: 19/09/2009–20/03/2011). Comparative genetic analysis revealed that clade A was associated with the *rsmD* gene, involved in DNA replication and repair; and clade B was associated with the *dcuB* gene, involved in the transport of C4-dicarboxylates into the cell. These genes were classified as pseudogenes in the other clade due to frameshifts. Two additional frameshifts and eleven non-synonymous SNPs in ten genes differenced between the clades. These genes varied in function (Additional file 5).

Linear regression modeling found that date of collection was not associated with the number of genes (p = 0.0635) or pseudogenes (p = 0.0501) (Additional file 6). Clades were not associated with different number of genes (p = 0.566) or pseudogenes (p = 0.539).

### United Kingdom patient phylogenetics

SNP analysis identified 122 non-recombinant SNPs amongst the 22 isolates collected from the United Kingdom patient. Phylogenetic modeling estimated that these isolates contained a median substitution rate of 1.96 × 10^–6^ substitutions site^−1^ year^−1^ (95% HPD interval: 1.51–2.44 × 10^–6^ substitutions site^−1^ year^−1^) and shared a common ancestor on 17/04/1999 (median, 95% HPD interval: 29/11/1997–23/05/2000) (Additional file 7). The sampled ancestor model identified three isolates (median, 95% HPD interval: 1–4) that represented ancestors of other isolates collected from the patient. Linear regression modeling found that date of collection was negatively associated with the number of genes (p = 0.0129) and positively associated with the number of pseudogenes (p = 0.00175) (Additional file 8).

### *Campylobacter* DNA replication and repair

DNA replication and repair mechanisms in *Campylobacter* involves 27 genes. 54 mutations were identified that differed between isolates collected from the New Zealand and United Kingdom patients (Additional file 9). The gene with the largest number of mutations was the *mutS* gene (n = 20).

### Long-term within human selection

Isolates from the New Zealand patient had a pangenome of 1722 genes and an accessory genome of 86 genes, whilst the isolates collected from the United Kingdom patient had a pangenome of 1743 genes and an accessory genome of 103 genes. Analysis of gene loss over the course of colonization identified six genes from the New Zealand patient isolates and five from the United Kingdom patient isolates that were stably-lost over time. However, neither these genes nor the rest of the accessory genome were associated with any functional group (Additional file 10).

**Fig. 3:**
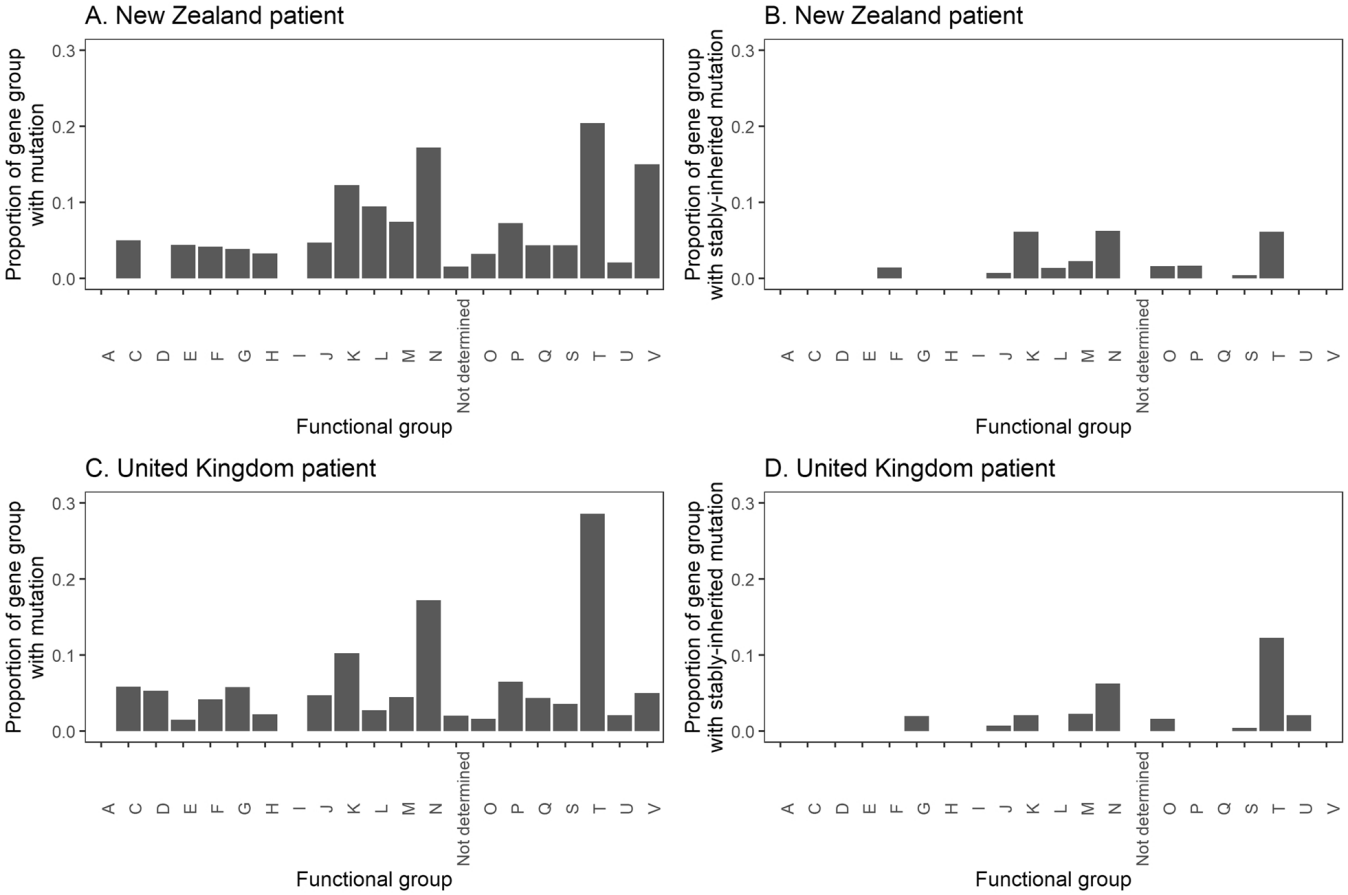
Bar plots of the proportion of genes of each functional group that contained non-synonymous SNPs or frameshifts (**A**, **B**) and stably-inherited SNPs or frameshifts (**C**, **D**) in the New Zealand (**A**, **C**) and United Kingdom (**B**, **D**) patients

The isolates collected from the New Zealand patient shared 134 core non-synonymous SNPs and 23 core frameshifts in 92 genes, whilst the isolates collected from the United Kingdom patient shared 88 core non-synonymous SNPs and 31 core frameshifts in 81 genes. Of these, 23 non-synonymous SNPs and two frameshifts from the New Zealand patient isolates, and 15 non-synonymous SNPs and six frameshifts from the United Kingdom patient isolates were stably-inherited. In both patients’ isolates, cell motility (COG group N) and signal transduction mechanisms (COG group T) were the functional groups with the highest proportion of genes containing these mutations (Fig. 3). In addition, some genes contained multiple non-synonymous SNPs and frameshifts (Additional file 11). The *porA* and *ccmL* genes were the only gene that had more than five of these mutations amongst isolates collected from both patients.

### 16S rRNA metabarcoding of bacterial species

**Fig. 4:**
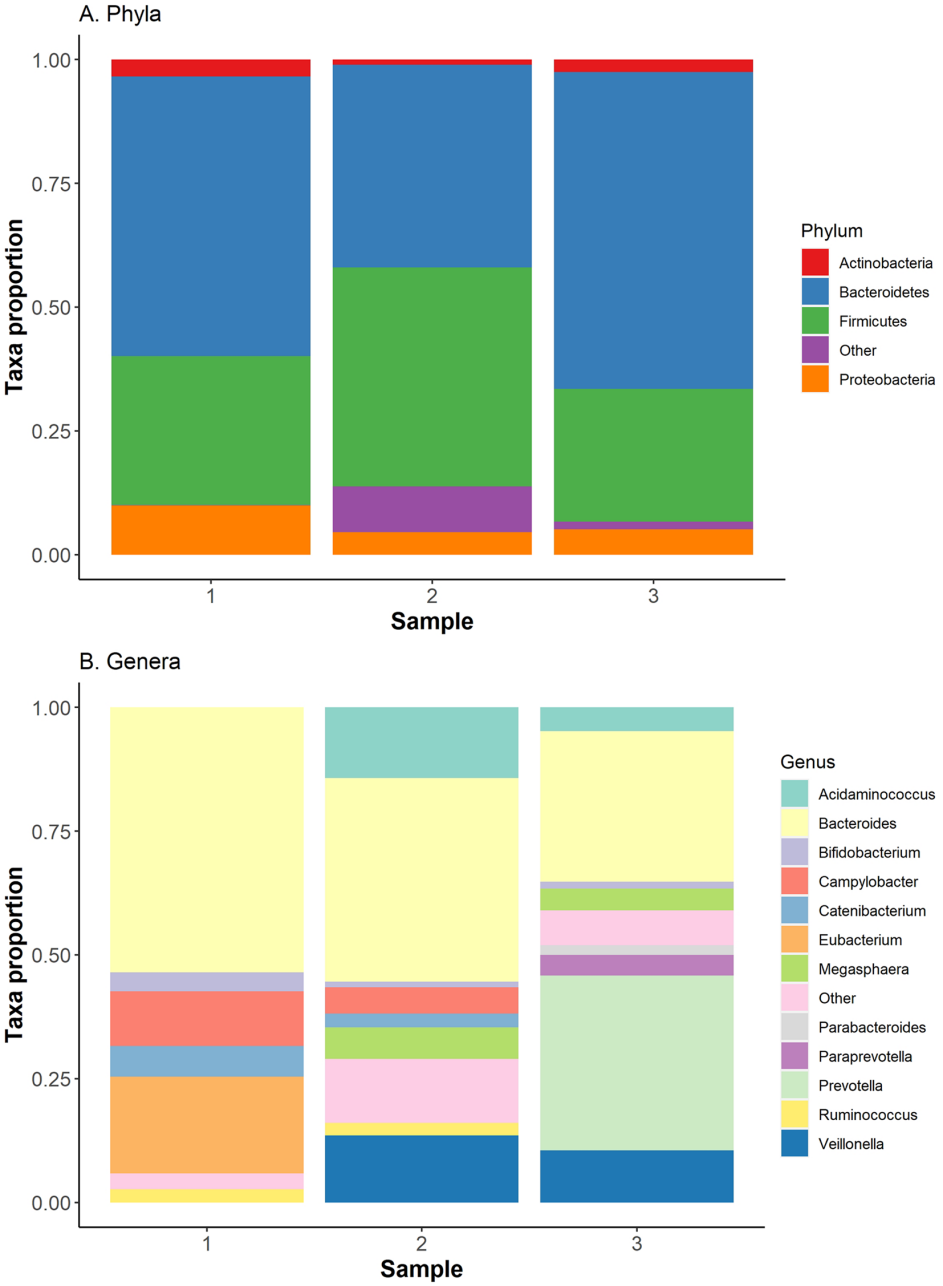
Bar plot of the proportion of the New Zealand patient’s microbiota, identified through 16S rRNA profiling, comprised of detectable bacterial phyla (**A**) and genera (**B**). Bacterial genera that represented less than one percent of the microbiota were placed in the ‘other’ category

The three fecal microbiota samples from the New Zealand patients displayed a large amount of variation in the proportion of each bacterial taxa present (Fig. 4). The *Campylobacter* concentrations were not directly correlated to the microbiota makeup, likely due to variations in the bacterial concentration of fecal samples.

### Biochemical and serological tests

**Fig. 5:**
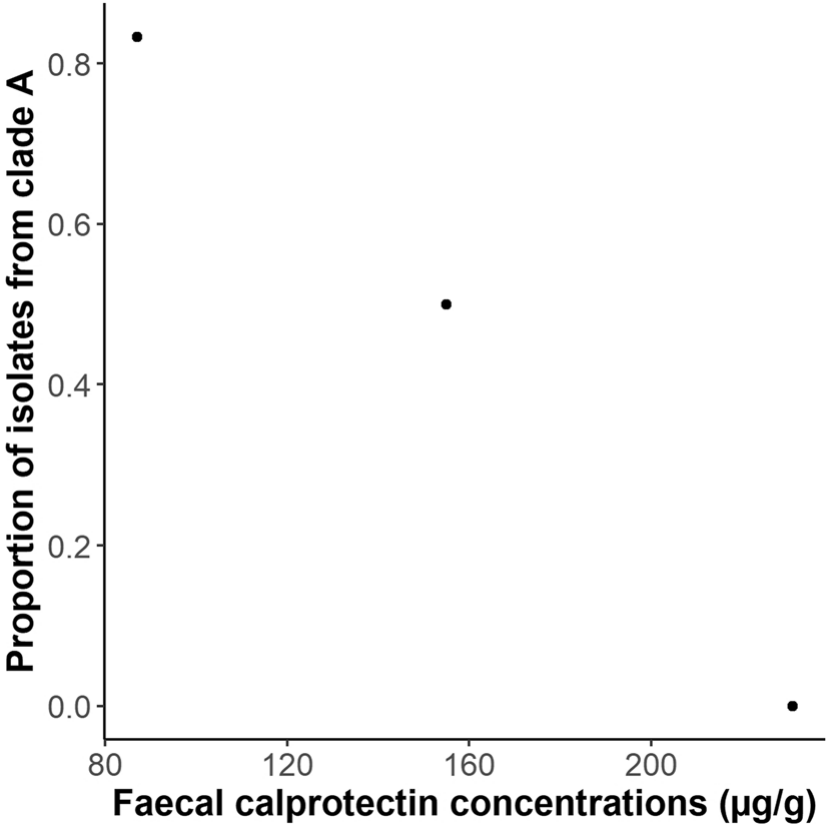
Scatterplots of the number of the proportion of isolates collected from clade A versus the fecal calprotectin concentration for three samples collected from the New Zealand patient

The New Zealand patient tested negative for anti-*Campylobacter* antibodies (Table 1), and had low immunoglobulin levels that were consistent with their immunosuppressive disease [13] and prior history [8]. The concentration of calprotectin was directly proportional to the number of isolates from clade B (Fig. 5). No other associations between the biochemical results, microbiota results, *Campylobacter* concentration and the proportion of each subpopulation were identified.

**Table 1:** Biochemical, serological and *Campylobacter* parameters for three samples collected over five months from the same patient

Sample	Measurement	1	2	3	Normal range
Date		03/09/2016	09/11/2016	21/02/2017	
Fecal consistency Calprotectin	Bristol stool scale µg/g	6–7 231	6–7 155	7 87	3–4 < 112
C-reactive protein	mg/L	0.84	1.45	1.13	< 5.0
IgA	g/L	< 0.2	< 0.2	< 0.2	0.7–4.0
IgG	g/L	3.9	4.5	4.7	7.0–16.0
IgM	g/L	< 0.2	< 0.2	< 0.2	0.4–2.3
anti-Campylobacter IgA	Titer	Negative	Negative	Negative	Negative
anti-Campylobacter IgG	Titer	Negative	Negative	Negative	Negative
anti-Campylobacter IgM	Titer	Negative	Negative	Negative	Negative
*Campylobacter* concentration	cfu/g	1.38 × 10^7^	1.48 × 10^7^	1.08 × 10^6^	0
Proportion *Campylobacter*		0.111	0.053	0.001	0
Clade A isolates		0/3	3/6	5/6	0

## Discussion

Long-term colonization of human patients places selective pressure on *Campylobacter*. Non-synonymous SNPs and frameshifts were found in multiple genes from *Campylobacter* isolates collected from the New Zealand and United Kingdom patients, especially in genes involved in motility and signal transduction mechanisms. Two genes were found that had more than five of these mutations in both patients: the *porA* gene that encodes the major outer membrane protein (MOMP) and the *ccmL* gene that encodes the multi-ligand binding chemoreceptor (CcmL). Previous work on isolates from the New Zealand patient demonstrated that they varied in motility, possibly to evade phagocytes [8]; whilst studies on *Campylobacter* collected from the same patients within 24-h demonstrated variation in motility and chemotaxis genes [14, 15]. This suggests that long-term colonization is selecting for changes in *Campylobacter* motility, signal transduction (particularly chemotaxis) and membrane proteins, possibly to evade host defenses.

Populations of bacteria are continuously accumulating mutations: canonically, if they are beneficial then those containing them will thrive and the mutation will increase in frequency, whilst if they are deleterious those containing them will struggle and the mutation will eventually disappear. However, Ramiro et al. [16] investigated *Escherichia coli* colonizing the gut of mice over 190 days and found that beneficial mutations increased in frequency but were not fixed in the population, and slightly deleterious mutations remained in the population for extended periods of time, possibly by the variable mouse gut buffering their deleterious effects. In this study, mutations were observed that were stably-inherited within the *Campylobacter* populations of the long-term patients, especially in those involved in cell motility and signal transduction. These *Campylobacter* were collected over longer time periods than Ramiro et al. experiments, possibly allowing these beneficial mutations to be fixed within the population or the *Campylobacter* were exposed to larger selective sweeps than the *E. coli*. However, a large number of mutations were observed in isolates that were not fixed within the *Campylobacter* population, suggesting that the human gastrointestinal tract allows for variation in many *Campylobacter* genes, but over time selective sweeps select for specific genetic variants. Nevertheless, we cannot rule out the possibility that many of the stably-inherited mutations were the result of “genetic hitchhiking” [17].

The isolates collected from the New Zealand and United Kingdom patients both belonged to ST45. However, within this sequence type the isolates from each patient were distantly related, allowing us to identify mutations specific to long-term colonization by comparing isolates from these patients to ST45 from other sources. Genetic comparisons identified one mutation associated with long-term patient colonization, T86I in the *gyrA* gene. This mutation is the most common cause of quinolone resistance in *C. jejuni* [11], and the New Zealand patient [8] and likely the United Kingdom patient [9] were prescribed quinolones, selecting for this mutation. A large number of other genes contained mutations in the New Zealand and United Kingdom patient isolates. However, the mutations either differed between isolates from each patient or were found in multiple other ST45 isolates. This suggests that apart from quinolone resistance, long-term human colonization selected for changes in particular genes, but not specific mutations.

Bacteria can adapt to environments by both gene loss and gene acquisition. Gene loss may increase the fitness of the bacteria by decreasing resource expenditure on unnecessary cellular processes [18], whilst gene acquisition may allow the bacteria to gain cellular processes to thrive in the environment [19]. Previous studies on long-term *Pseudomonas aeruginosa* cystic fibrosis (CF) [20] and *Salmonella enterica* [21] infections found that gene loss outweighed gene acquisition over the course of these infections. However, Bayjaynov et al*.* [22] investigated long-term colonizing *E. faecium* strains and found similar amounts of gene loss and gene acquisition. In our study, several genes were identified that were stably-lost over the course of colonization in the New Zealand and United Kingdom patients, and there was some evidence of the total number of genes decreasing over time. However, no functional group was overrepresented in the accessory genome, suggesting that genome reduction is not the main way *Campylobacter* adapts to long-term colonization. Gene loss could have occurred between acquisition and isolation of the first isolate, but there was no evidence that the long-term patients had smaller number of genes compared to the other human ST45 isolates. This is a similar observation to long-term *E. faecium* colonization rather than to *P. aeruginosa* or *S. enterica* infections, namely evidence of similar amounts of gene loss and gene gain. Further research is required to determine if this is due to the different biology of these bacteria, different environments, or different types of infection.

In the New Zealand patient, we identified two genetically distinct clades or subpopulations of *Campylobacter*. In CF infections, divergence of *P. aeruginosa* subpopulations has been reported prior to infection [23] and within the host [24]. Analysis of long-term *E. faecium* colonization has demonstrated the presence of multiple lineages within the gastrointestinal tract of humans, but these lineages diverged prior to colonization [22]. Phylogenetic analysis suggests that the two *Campylobacter* subpopulations diverged between 2009 and 2011, after the first isolate was collected from the patient in 2006, indicating that the subpopulations diverged within the host. There is evidence of multiple subpopulations coexisting prior to this divergence in the New Zealand patient, and in the isolates collected from the United Kingdom patient, but these patients were not sampled frequently during these time periods, preventing further analysis of these subpopulations.

The competitive exclusion principle states that no two species can occupy the same niche [25]. Accordingly, the subpopulations of *Campylobacter* found within the patient are likely to have distinct niches. In CF patients, the presence of multiple subpopulations has been attributed to the bacterium accumulating mutations and phenotypically adapting to different environments within the human lung [26]. There is evidence of this when looking at the genetic differences between the two *Campylobacter* lineages found within the human host, specifically the *dcuB* gene that encodes a fumarate/succinate active antiporter under low oxygen conditions that can also import aspartate [27]. Succinate concentrations in the gastrointestinal tract have been shown to increase during gut dysbiosis [28] and inflammation [29], but it remains to be determined if these conditions affect fumarate or aspartate concentrations. The New Zealand patient’s gastrointestinal tract showed a large amount of variation in inflammation plus variation in the microbiota composition and these may be influencing the concentration of succinate and possibly other metabolites. The concentration of calprotectin was negatively associated with the proportion of isolates made up of the lineage missing *dcuB*, suggesting that the two lineages could have emerged in response to the variation in gastrointestinal inflammation and microbiota disruptions. The *dcuA* gene is a fumarate/aspartate:H^+^ symporter and 90% of isolates from the *dcuB*-negative clade contained the L212F mutation in the *dcuA* gene, whilst all isolates belonging to the *dcuB*-containing clade contained the S148F mutation in this gene. However, it is unclear how these mutations affect the function of this transporter. DcuA and DcuB work in close association with the cytoplasmic-facing fumarate reductase FrdABC, but no mutations were found in the *frdA, frdB* or *frdC* genes [30]. Nevertheless, the absence of DcuB would prevent correct stoichiometric exchange of succinate and fumarate that allow FrdABC to function. *Campylobacter* also has a unidirectional fumarate reductase that cannot oxidize succinate, MfrABE, that operates independently of DcuA and DcuB, as it is periplasm-facing [31]. For the clade with an intact *dcuB* gene, three out eighteen of the isolates contained a truncated *mfrA* gene due to a frameshift. It has been shown that FrdABC is the major contributor to fumarate reduction but that MfrABE is required for full fitness when the bacteria rely on fumarate respiration to conserve energy [31]. Taken together, these observations suggest that different clades will have distinct contributions of Dcu/Frd versus Mfr mediated fumarate respiration during colonization. The role of DcuA and DcuB in aspartate uptake in low oxygen intestinal niches may also be physiologically important. Further work on the concentrations of gastrointestinal aspartate, succinate and fumarate in the patient and how this relates to the different lineages is required to determine if gastrointestinal inflammation is creating distinct niches where certain metabolites are not required. However, multiple other mutations were found amongst the two lineages and we cannot rule out the possibility that the mutations associated with *dcuB* were genetic hitchhikers.

*C. jejuni* ST45 is one of the most frequently isolated strains of *Campylobacter* collected from humans, domestic animals and the environment [32–34]. It is regarded as a ‘generalist’ strain, as it has been isolated from multiple host species and environments [35, 36], and has demonstrated frequent host switching [37]. We found clades of ST45 that consisted of isolates collected from different sources, supporting ST45 as a generalist strain, but also found some clades that only consisted of isolates from single sources and many clades that only consisted of a single non-human source. Further analysis of non-human ST45 isolates is required to determine the extent that ST45 is a “generalist” strain.

The human microbiota is affected by disease [38], diet [39] and genetics [40]. Youmans et al. [38] investigated the microbiota of individuals with traveler’s diarrhea and found that regardless of cause, diarrheic samples contained a high *Prevotella*-to-*Bacteroides* (P/B) ratio. This observation is supported in this study, where the third diarrheal sample collected from the patient was higher on the Bristol scale (softer) and had a higher P/B ratio. Braun et al. [41] investigated the microbiota of healthy individuals and hospitalized patients suspected of infectious diarrhea, and found diarrhea patients were associated with an increased abundance of *Proteobacteria*. The first diarrheal sample collected from the patient had the highest *Proteobacteria* proportion, but this is because it has the highest *Campylobacter* proportion which made up most of the *Proteobacteria* in the microbiota of this sample. Zhuang et al. [42] found that diarrhea brought on by irritable bowel syndrome resulted in increased *Bacteroidetes* and decreased *Firmicutes*, such as with the first two samples. However, the amount of *Prevotella* and *Proteobacteria* in the microbiota is also affected by diet [39, 43]. Further microbiota samples and dietary information from the time of collection are required to investigate the role of disease and diet on the patient’s microbiota.

The sampled ancestor model identified multiple isolates from the United Kingdom patient that represented ancestors to other isolates collected from this patient, but it detected no likely ancestors amongst the New Zealand patient’s isolates. This suggests that the population of *Campylobacter* was more diverse within the New Zealand patient than within the United Kingdom patient, and therefore sampled isolates were less likely to represent an ancestral state. However, *Campylobacter* within these patients have likely undergone multiple bottlenecks, especially with antimicrobial therapies [8, 9], and we cannot rule out the possibility that the United Kingdom patient was sampled during several of these bottlenecks when the genetic diversity was smaller, whilst the New Zealand patient was not.

*Campylobacter* collected from the New Zealand patient had a substitution rate twice that of those collected from the United Kingdom patient, with no overlap between 95% HPD intervals. Multiple differences were found in genes involved in DNA replication and repair between isolates collected from these patients. The *mutS* gene had the largest number of differences, but knockouts in this gene in the closely related bacterium, *Helicobacter pylori*, are not associated with increased substitution rates [44]. Mutations in *mutY* have been associated with faster substitution rates in *Campylobacter* [45, 46], but isolates collected from the long-term patients had identical *mutY* genes. Further work is required to determine the effects of other mutations in DNA replication and repair genes on the substitution rate of *Campylobacter*.

The New Zealand patient’s gastrointestinal health, amount of inflammation and immunosuppression varied significantly between the three samples obtained, as indicated by the variation in serum IgA, IgG, IgM and CRP concentrations, and fecal calprotectin concentration. Apart from the association between the proportion of each clade isolated and fecal calprotectin concentrations, no other associations were identified between these markers, the fecal *Campylobacter* concentration, microbiota constitution, and the proportion of each clade isolated. The variation in these biochemical tests over these five months does suggest that the patient was undergoing changes in gastrointestinal health and immunosuppression.

An original objective of this research was to determine if *Campylobacter* was contributing to the patient’s diarrheal episodes or simply colonizing the patient. The lack of evidence for anti-*Campylobacter* antibodies suggests that the patient had not mounted an immune response against the *Campylobacter*. However, most studies on *Campylobacter* serology have focused on acute infections, rather than the possible chronic infection described here. In addition, most serological tests have a high false-negative rate and for those individuals that do seroconvert the antibody titer quickly decreases after a few months [47, 48]. Studies on acute Guillain-Barré syndrome, a disease often triggered by *Campylobacter* infections have found up to 80% of cases display serological evidence of *Campylobacter*, but it is unclear whether the negative cases were triggered by *Campylobacter* or other infections [49], and false positives have been observed [50]. Regarding the New Zealand patient, this could be explained by a number of scenarios including: the *Campylobacter* was not the cause of any pathology and had not been presented to the immune system or triggered an immune response; the *Campylobacter* contributed to the diarrheal episodes but the patient was unable to form an immune response sufficient to remove the bacteria or be detected using the serological method described.

## Conclusions

In this study we investigated the genomes of *Campylobacter* from two patients that were both colonized for over a decade and found that multiple selective pressures were imposed on the *Campylobacter*. There was some evidence of genome degradation, but more evidence that *Campylobacter* adapted through the accumulation of non-synonymous SNPs and frameshifts in genes involved in cell motility, signal transduction and the major outer membrane protein, possibly to evade host defenses. However, we also found that the *Campylobacter* collected from the patients differed in substitution rates and diversity as evidenced by sampled ancestral states, although the reason for these differences remains unclear. Through 16S rRNA metabarcoding and biochemical markers we demonstrated that the New Zealand patient displayed a large amount of variation in their microbiome, inflammation and immunosuppression over five months, and that the *Campylobacter* collected from the patient could be divided into two subpopulations, the proportion of which correlated with the amount of gastrointestinal inflammation. This suggests that subpopulations of *Campylobacter* evolve within the gastrointestinal tract to adapt to changing environments. Overall, this study demonstrates how genomics, phylogenetics, 16S rRNA metabarcoding and biochemical markers can provide insight into how *Campylobacter* adapts to changing environments within human hosts.

## Methods

### Sample collection and storage

The previously described New Zealand patient [8] was invited to take part in a year-long study to investigate the effect of long-term *Campylobacter* colonization. Every month serum and fecal samples were collected from the patient. The Bristol fecal chart was used to grade the consistency of the fecal sample [51]. Approximately 100 mg of fecal sample was set aside for immediate *Campylobacter* culturing. The rest of the fecal sample and the serum sample were stored at −80 °C until all samples were collected and were then further analyzed in a single batch.

### *Campylobacter* quantification

Approximately 100 mg of each fecal sample was resuspended in 9.9 ml of phosphate-buffered saline (PBS) (prepared in house). The reconstituted solution was serially diluted three times by adding 1 ml of sample to 9 ml of PBS, before 100 µl of each dilution was spirally plated onto modified charcoal-cefoperazone-deoxycholate agar (mCCDA) (Fort Richard Laboratories, Auckland, New Zealand) in duplicate. For plates that contained 50–500 colonies, the number of colonies was quantified and used to calculate the concentration of *Campylobacter* in the fecal samples. From each sample, 3–6 *Campylobacter* colonies underwent genomic DNA extractions and were whole genome sequenced as previously described [8] using the Illumina MiSeq platform (Illumina, San Diego, California, United States).

### Serum biochemistry and serology

The SERION ELISA classic *Campylobacter jejuni* IgA, IgG and IgM tests (Institut Virion\Serion GmbH, Wurzburg, Germany) were used to calculate the amount of anti-*Campylobacter* immunoglobulin A (IgA), immunoglobulin G (IgG) and immunoglobulin M (IgM) in the serum according to the manufacturer’s instructions. An aliquot of each serum sample was sent to MedLab Central (Palmerston North, New Zealand), who quantified the total serum IgA, IgG and IgM, and the serum inflammation marker, C-reactive protein (CRP), on a Cobas 8000 modular analyzer with the c702 module (Roche, Basel, Switzerland).

### Fecal biochemistry

An aliquot of each fecal sample was sent to Canterbury Health Laboratories to quantify the amount of the gastrointestinal inflammation marker, calprotectin, using the Calpro Calprotecin ELISA (Calpro AS, Lysaker, Norway).

### 16S rRNA gene-based microbiota analysis

The DNeasy PowerSoil Kit (Qiagen, Hilden, Germany) was used to extract DNA from an aliquot of each fecal sample. PCR was used to amplify the 16S V3-V4 region of bacterial DNA in the extracts and libraries were made from the 16S rRNA gene amplicons using a single-step PCR with barcoded primers [52]. The libraries were sequenced on an Illumina MiSeq as 2 × 250 paired-end reads. The reads were investigated with the MG-RAST pipeline [53].

### ST45 pan genome analysis

The accession numbers of available *C. jejuni* ST45 genome sequences were identified by searching the PATRIC database [54] and the Sequence Read Archive (SRA; https://www.ncbi.nlm.nih.gov/sra) for “Campylobacter”, “ST45” and “Paired-end reads”. The raw reads of these sequences were downloaded and trimmed along with those collected from the patient using Trimmomatic v0.39 [55]. ARIBA v2.14.4 [56] was used to determine the sequence type of these reads using the *C. jejuni* multi-locus sequence type (MLST) [57], and all isolates that were identified as ST45 were taken forward for further analysis (Additional file 1).

The trimmed reads of all ST45 isolates were assembled using Spades v3.14.0 [58], and the assemblies produced were analyzed using Quast v5.0.2 [59] and CheckM v1.1.2 [60]. All assemblies that contained more than 300 contigs greater than 500 bp or had more than 50 duplicate genes were regarded as potentially contaminated or not sequenced at a high enough read depth and were not analyzed further.

The assemblies that passed QC were annotated using Prokka v1.12 [61] and the pan-genome evaluated using Roary v3.11.2 [62] with a 95% identity cut-off. Genes that were found in over 95% of the isolates were classified as part of the core genome. A maximum likelihood tree was constructed from single nucleotide polymorphisms (SNPs) in the core gene alignment using RAxML v8.2.11 [63]. Annotation, pan-genome evaluation and phylogenetics were repeated with reference genome NC_002163 [64] to root the tree. TreeCluster v1.0.1 [65] was used to predict clades in the maximum likelihood tree.

RFPlasmid v0.0.16 [66] was run on all assemblies with the “*Campylobacter*” database. Contigs with > 0.6 votes for plasmid were classified as “plasmid-associated”. Abricate v1.0.1 (https://github.com/tseemann/abricate) with the ResFinder [67] and virulence finder database (VFDB) [68] were used to search plasmid-associated contigs for AMR and virulence genes. Mob-typer [69] v3.0.0 was used to determine the mobility of the plasmid-associated contigs.

The ResFinder database, VFDB database, PlasmidFinder database [70], and a custom database consisting of all genes clustered by Roary were used with ARIBA to identify the presence of acquired AMR genes, virulence genes, plasmids and pseudogenes, respectively, in the ST45 genomes. Pseudogenes were identified from the custom database using the method described by Mather et al*.* [71]. Pseudogenes were analyzed if the original gene or pseudogene were found in 95% of isolates. To identify mutations associated with macrolide or fluoroquinolone resistance, an ARIBA database was formed from the 23S rRNA and *gyrA* genes of *C. jejuni* NCTC 11168 (NC_002163).

Genes and pseudogenes were classified as “long-term patient-specific” if they were found in 95% of isolates collected from the New Zealand and United Kingdom patients and fewer than 5% of ST45 isolates collected from other sources or other human sources. EggNOG v4.5.1 [72] was used to predict the function of the genes. Linear regression models were used to model the total number of genes and pseudogenes with source and country as the explanatory variables. Isolates where the country or source were unknown were excluded from the model, as were singletons. The long-term patients contained multiple isolates for a sample, so the mean number of genes and pseudogenes for these patients was modeled. Partial-F tests were used to determine if these variables had a significant effect on the model.

For the long-term New Zealand patient, linear regression models were used to model the total number of genes and pseudogenes with date of collection and clade as the explanatory variables. For samples where multiple isolates were collected, the mean number of genes and pseudogenes for each clade was calculated. Partial-F tests were used to determine if inclusion of clades had a significant effect on the model.

The trimmed reads of the genome sequences collected from the New Zealand patient and those belonging to the most closely related clade, were aligned to the ST45 reference genome NC_022529 using Snippy v4.4.5 (https://github.com/tseemann/snippy). Areas of putative recombination were removed from the full alignments produced using Gubbins [73]. RAxML was used to form a maximum likelihood tree from the non-recombinant SNPs identified.

### Within-patient ST45 phylogenetics

The trimmed reads of all genomes that were collected from the New Zealand patient were aligned to the ST45 reference genome NC_022529 using Snippy and areas of putative recombination were removed from the full alignments produced using Gubbins. To determine if there was a clock signal, IQ-TREE v1.6.12 [74] was used to form a maximum likelihood tree from the identified non-recombinant SNPs and TempEst v1.5.3 [75] was used to test for temporal signal.

The non-recombinant SNPs were exported into BEAUti v2.5 to create an Extensive Markup Language (XML) file for BEAST v2.5 [76]. The ST45 reference genome NC_022529, consists of 584,548 adenine, 254,724 cytosine, 253,882 guanine and 576,175 thymine nucleotides; these nucleotide proportions (adjusted for the SNPs) were added as non-varying sites to keep the model representative of ST45 genomes and to calculate the substitution rate. bModelTest [77] was used to choose the substitution model (Supplementary Material). Multiple molecular clock (strict, random [78] and uncorrelated relaxed [79]) and tree models (constant and Extended Bayesian Skyline) [80]) were trialled for 100 million steps. Nested sampling [81] was used to select the model. A Generalized Time Reversible (GTR) [82] model was used to model nucleotide substitutions, an Extended Bayesian Skyline model was used to model the effective population size, and a strict clock model was used to model the molecular clock and was calibrated by the tip dates. A uniform prior was placed on the substitution rate that it would not exceed 10^–4^ substitutions site^−1^ year^−1^ nor fall below 10^–8^ substitutions site^−1^ year^−1^ [83], and due to the large number of constant sites added, the proportion of invariant sites was assumed to be zero. The XML file was run in BEAST in three separate chains with different starting seeds of 50 million steps each, before LogCombiner v2.5 was used to combine the runs with a 10% burn-in removed. Tracer v1.6 [84] was used to visualize the results.

TreeAnnotator v2.5 was used to form a maximum clade credibility tree of the SNP dataset. Evolview v2 [85] was used to visualize and edit the tree. The Sampled Ancestor (SA) v2.02 model [86] was also run in BEAST2 to determine the likelihood that any of the isolates collected from the patient represented ancestral states.

### Second long-term *Campylobacter* patient

The ST45 isolates downloaded included 22 genomes from an immunosuppressed long-term *Campylobacter* patient from the United Kingdom [9]. The phylogentic analyses outlined above were also performed on these isolates.

### Phase variation and non-synonymous SNPs

To investigate phase variation, Tatajuba v1.0.2 [87] was used to align the trimmed reads of all the ST45 isolates to the reference genome, NC_022529, and identify tracts that differed in size between the isolates. A cut-off of 0.95 was used when comparing frameshifts between different groups of ST45 isolates to account for small proportions of sequencing errors. To investigate non-synonymous SNPs, Snippy was used to align the trimmed reads of all the ST45 isolates to the same reference genome and identify SNPs that resulted in amino acid changes. Frameshifts and SNPs from long-term patient isolates were compared to ST45 isolates from other sources or other human sources as above to determine if they were associated with long-term patients.

### Replication genes

To identify possible reasons for variation in substitution rates between isolates from each long-term patient, KEGG (https://www.genome.jp/kegg/) was searched for all genes involved in *Campylobacter* DNA replication and repair. An ARIBA database was created from these genes and used to search all the ST45 isolates for their presence and mutations in these genes. Mutations found in > 95% of isolates in one patient and < 5% of isolates in the other were classified as associated with a patient.

### Stably-inherited genetic changes

To identify genetic changes that were stably-inherited, non-synonymous SNPs, frameshifts and gene loss were identified in the *Campylobacter* colonizing the long-term patients that were found in all isolates collected after a time point and not in any prior to this time point. Genetic changes that occurred between the collection of the first and second isolates, and last and second-to-last isolates were ignored as their stability was based on one isolate.

## Supplementary Information

**Additional file 1.** Genomic information and metadata on ST45 isolates investigated.

**Additional file 2. **ST45 pangenome linear regression modeling.

**Additional file 3. **ST45 clade 18 analysis.

**Additional file 4. **New Zealand patient phylogenetic analysis.

**Additional file 5. **New Zealand patient clade gene analysis.

**Additional file 6. **New Zealand patient gene number modeling.

**Additional file 7. **United Kingdom patient phylogenetic analysis.

**Additional file 8. **United Kingdom patient gene number modeling.

**Additional file 9. **Tables of the proportion of DNA replication genes found in long-term *Campylobacter* patients and other ST45 isolates (**Table S4**), and the mutations in *Campylobacter* DNA replication genes associated with isolates collected from long-term patients (**Table S5**).

**Additional file 10. **Long-term patients’ pangenome function analysis.

**Additional file 11. **Long-term patients’ gene variation function analysis.

## Data Availability

The reads of the *Campylobacter* genomes and the 16S rRNA sequences were uploaded to NCBI under the Accession Numbers PRJEB24941 and PRJNA605845, respectively.

## Abbreviations

AMR: Antimicrobial resistance
*C. jejuni*: *Campylobacter jejuni*
CF: Cystic fibrosis
COG: Clusters of orthologous groups
CRP: C-reactive protein
CVID: Common variable immune deficiency
*E. faecium*: *Enterococcus faecium*
HPD: Highest posterior density
IgA: Immunoglobulin A
IgG: Immunoglobulin G
IgM: Immunoglobulin M
GTR: Generalized time reversible
mCCDA: Modified charcoal cefoperazone deoxycholate agar
MLST: Multi-locus sequence typing
MOMP: Major outer membrane protein
*P. aeruginosa*: *Pseudomonas aeruginosa*
P/B: *Prevotella*-To-*Bacteroides*
PBS: Phosphate buffered saline
*S. enterica*: *Salmonella enterica*
SA: Sampled ancestor
SNP: Single nucleotide polymorphism
SRA: Sequence read archive
ST: Sequence type
VFDB: Virulence finder database
XML: Extensible markup language
