# Supplementary material for: Genomic adaptations of Campylobacter jejuni to long-term human colonization
Source: Gut Pathog. 2021 Dec 10;13:72. doi: 10.1186/s13099-021-00469-7 (PMC8665580; doi:10.1186/s13099-021-00469-7)
Supplement: Supplementary file 2 — Additional file 2. ST45 pangenome linear regression modeling. [file 13099_2021_469_MOESM2_ESM.docx]

**ST45 pangenome linear regression modeling**

The number of genes and pseudogenes identified in the ST45 isolates showed a large amount of variation amongst isolates from different sources and countries (Figure S1). They were modelled using linear regression analysis with source and country as the explanatory variables (Figures S2-S3). Isolates where the country or source were unknown were excluded from the model, as were singletons. The long-term patients contained multiple isolates for a sample, so the mean number of genes and pseudogenes for these patients was modelled (Figure S4). Partial-F tests were used to determine if source and country significantly affected the models.


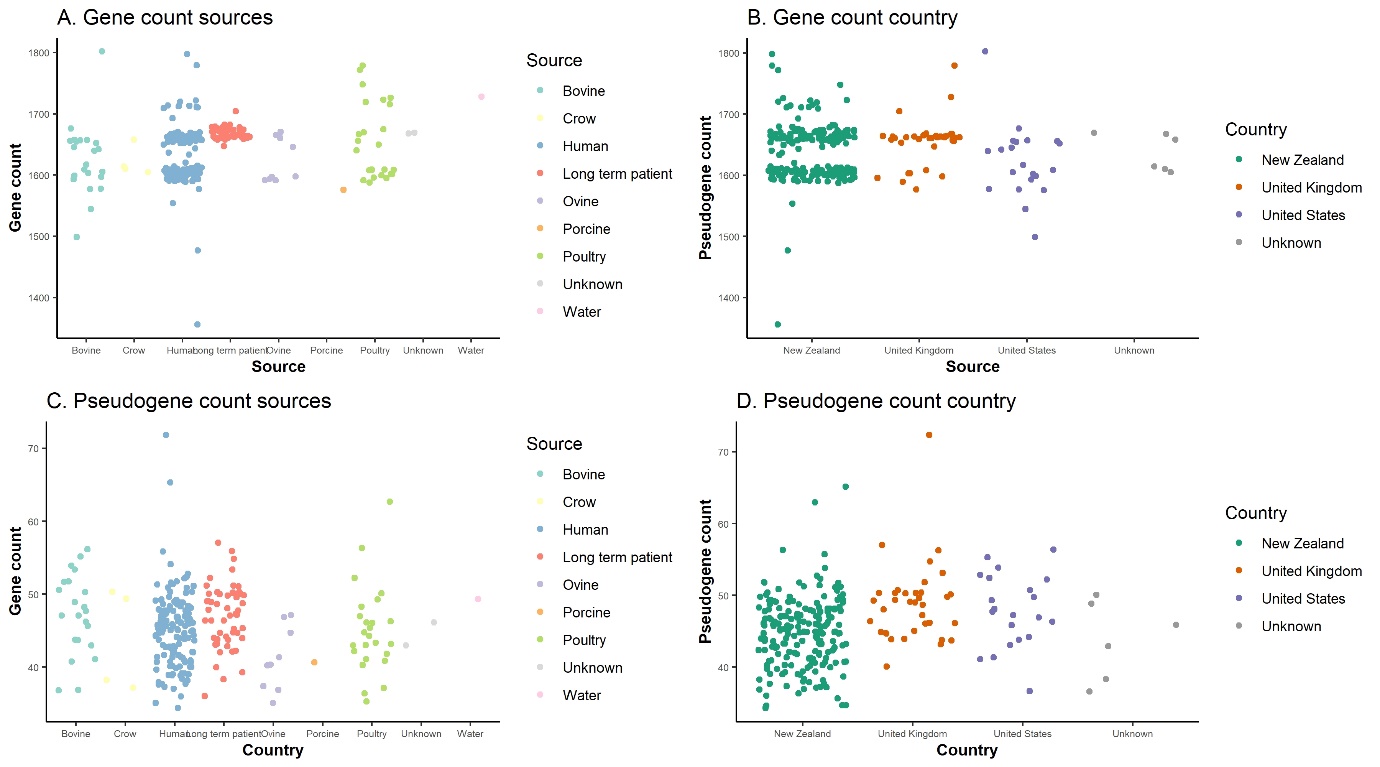


**Figure S1.** Jitter plots of the number of genes (A and B) and pseudogenes (C and D) found amongst ST45 isolates separated by source (A and C) and country (B and D).


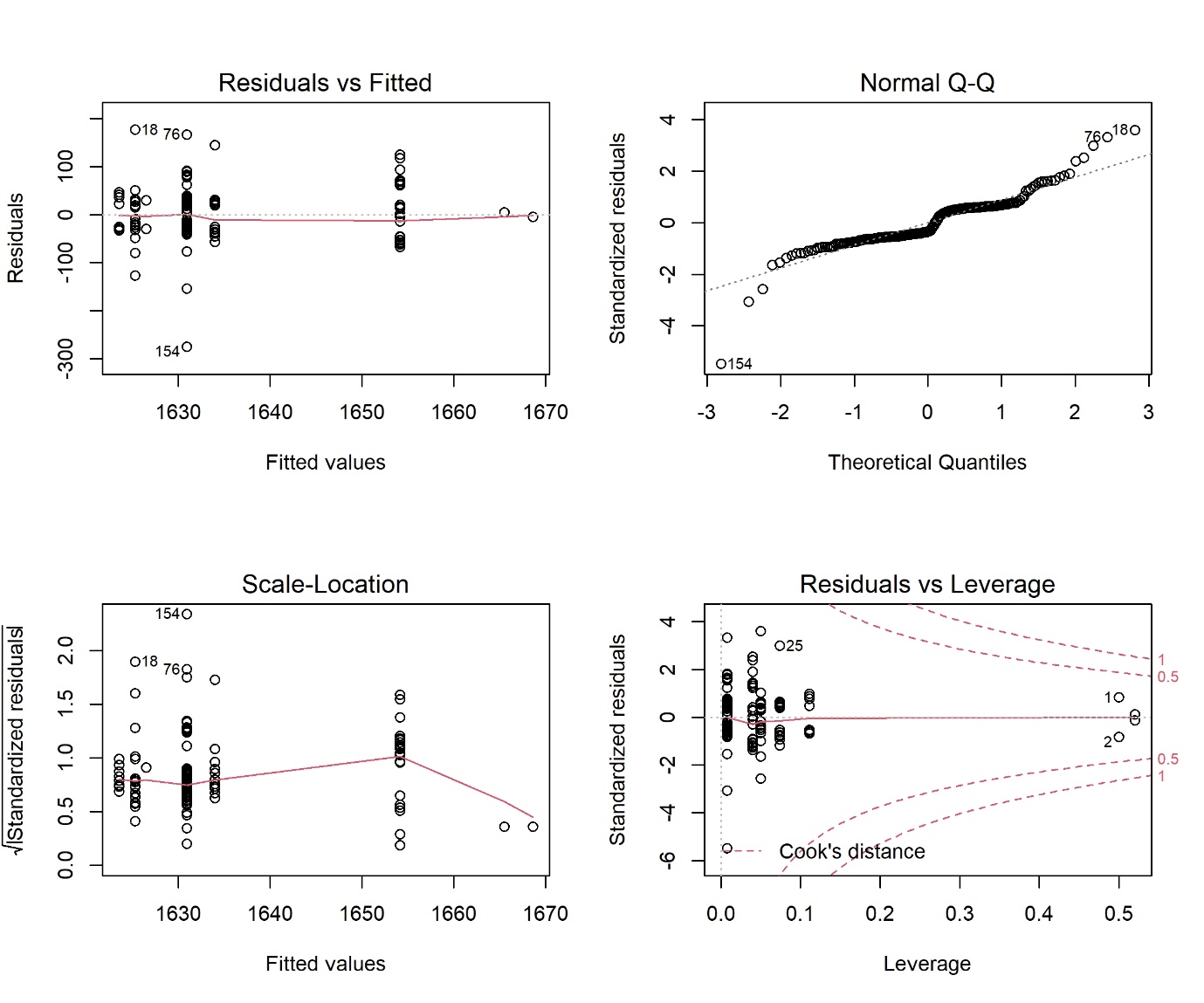


**Figure S2**. Regression plots of the linear regression model used to model the number of genes from ST45 isolates.


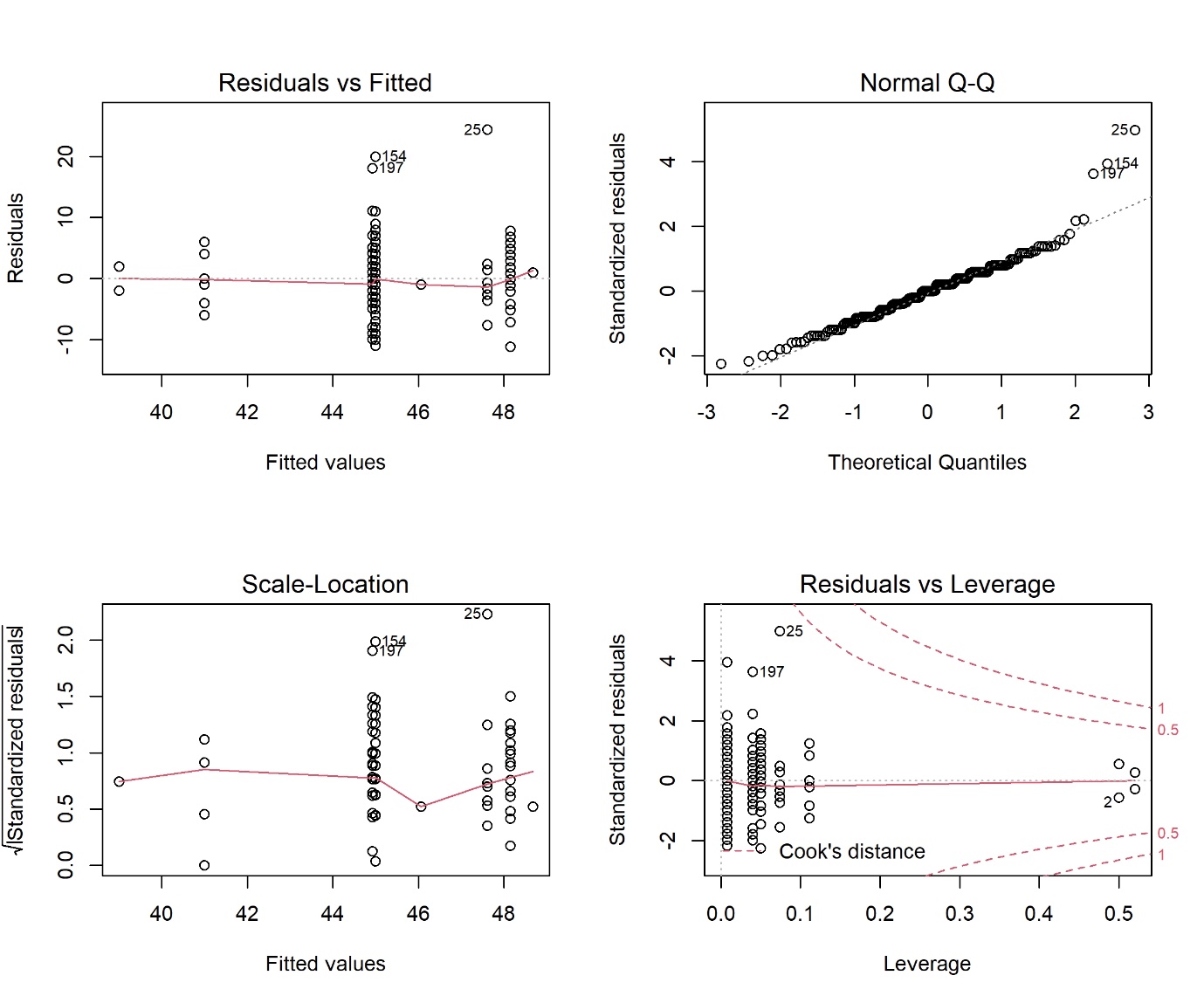


**Figure S3**. Regression plots of the linear regression model used to model the number of pseudogenes from ST45 isolates.


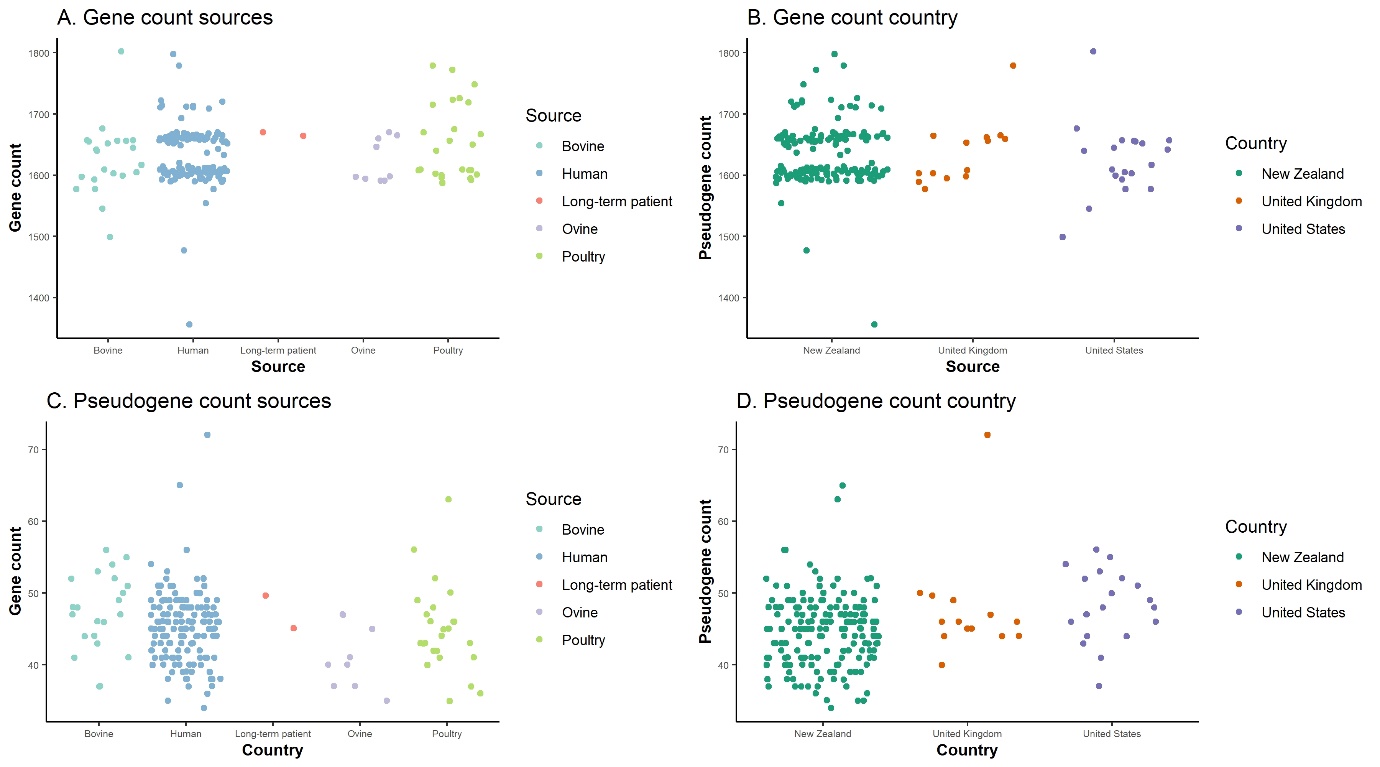


**Figure S4.** Jitter plots of the number of genes (A and B) and pseudogenes (C and D) found amongst ST45 isolates separated by source (A and C) and country (B and D) that were modelled.

The linear regression models explained very little of the variance in gene (R^2^ = 0.00185) and pseudogene (R^2^ = 0.06277) numbers. Gene numbers were not significantly affected by source (p = 0.2216) or country (p = 0.9765). Pseudogenes were not significantly affected by source (p = 0.101) but were by country (p = 0.0114).

**ST45 gene linear regression analysis**

Genes = factor(Source) + factor(Country)

Residuals

| Minimum | 1Q | Median | 3Q | Maximum |
| --- | --- | --- | --- | --- |
| -274.9 | -29.22 | -16.94 | 30.06 | 176.7 |

| Coefficient | Estimate | Standard error | t-value | p-value |
| --- | --- | --- | --- | --- |
| Intercept | 1627 | 35.62 | 45.66 | <2 x 10^-16^ |
| Source:Human | 4.436 | 35.89 | 0.124 | 0.902 |
| Source:Long-term patient | 39.06 | 50.89 | 0.768 | 0.444 |
| Source:Ovine | -2.944 | 39.38 | -0.075 | 0.940 |
| Source:Poultry | 27.70 | 37.02 | 0.748 | 0.455 |
| Country:United Kingdom | 3.094 | 14.35 | 0.216 | 0.829 |
| Country:United States | -1.200 | 37.36 | -0.032 | 0.974 |

| Statistic | Value |
| --- | --- |
| Residual standard error | 50.38 on 196 degrees of freedom |
| Multiple R^2^ | 0.0315 |
| Adjusted R^2^ | 0.00185 |
| F-statistic | 1.062 on 6 and 196 degrees of freedom |
| p-value | 0.3866 |

**ST45 gene source parital-F test**

| Model | Residual degrees of freedom | RSS | Degrees of freedom | Sum of squares | F-value | p-value |
| --- | --- | --- | --- | --- | --- | --- |
| ST45 gene model | 196 | 497452 |  |  |  |  |
| ST45 gene model – minus source | 200 | 512092 | -4 | -14640 | 1.442 | 0.2216 |

**ST45 gene country parital-F test**

| Model | Residual degrees of freedom | RSS | Degrees of freedom | Sum of squares | F-value | p-value |
| --- | --- | --- | --- | --- | --- | --- |
| ST45 gene model | 196 | 497452 |  |  |  |  |
| ST45 gene model – minus country | 198 | 497573 | -2 | -120.7 | 0.0238 | 0.9765 |

**ST45 pseudogene linear regression analysis**

Pseudogenes = factor(Source) + factor(Country)

Residuals

| Minimum | 1Q | Median | 3Q | Maximum |
| --- | --- | --- | --- | --- |
| -11.15 | -3.612 | 0.0073 | 3.007 | 24.39 |

| Coefficient | Estimate | Standard error | t-value | p-value |
| --- | --- | --- | --- | --- |
| Intercept | 39.00 | 3.597 | 10.84 | <2 x 10^-16^ |
| Source:Human | 5.993 | 3.624 | 1.654 | 0.0998 |
| Source:Long-term patient | 7.057 | 5.138 | 1.373 | 0.1712 |
| Source:Ovine | 2.000 | 3.977 | 0.503 | 0.6156 |
| Source:Poultry | 5.920 | 3.738 | 1.584 | 0.1149 |
| Country:United Kingdom | 2.620 | 1.448 | 1.809 | 0.0720 |
| Country:United States | 9.150 | 3.772 | 2.425 | 0.0162 |

| Statistic | Value |
| --- | --- |
| Residual standard error | 5.087 on 196 degrees of freedom |
| Multiple R^2^ | 0.0906 |
| Adjusted R^2^ | 0.06277 |
| F-statistic | 3.255 on 6 and 196 degrees of freedom |
| p-value | 0.00451 |

**ST45 pseudogene source parital-F test**

| Model | Residual degrees of freedom | RSS | Degrees of freedom | Sum of squares | F-value | p-value |
| --- | --- | --- | --- | --- | --- | --- |
| ST45 pseudogene model | 196 | 5072 |  |  |  |  |
| ST45 pseudogene model – minus source | 200 | 5275 | -4 | -203.6 | 1.967 | 0.101 |

**ST45 pseudogene country parital-F test**

| Model | Residual degrees of freedom | RSS | Degrees of freedom | Sum of squares | F-value | p-value |
| --- | --- | --- | --- | --- | --- | --- |
| ST45 pseudogene model | 196 | 5072 |  |  |  |  |
| ST45 pseudogene model – minus country | 198 | 5038 | -2 | -236.9 | 4.577 | 0.0114 |
