# Supplementary material for: Genomic adaptations of Campylobacter jejuni to long-term human colonization
Source: Gut Pathog. 2021 Dec 10;13:72. doi: 10.1186/s13099-021-00469-7 (PMC8665580; doi:10.1186/s13099-021-00469-7)
Supplement: Supplementary file 3 — Additional file 3. ST45 clade 18 analysis. [file 13099_2021_469_MOESM3_ESM.docx]

**ST45 clade 18 analysis**

Phylogenetic analysis identified two isolates from New Zealand human clinical cases that were closely related to those from the long-term New Zealand patient and shared identical AMR genes and mutations (Figure S5). We could not rule out the possibility that these two isolates were also from the same long-term New Zealand patient, but not identified as so from the laboratory records. Alternatively, there may have been a common source of infection in New Zealand for the long-term patient was consistently exposed to it. This is unlikely given that the *Campylobacter* developed resistance to the antimicrobial agents the patient was prescribed [1] and the high correlation between the proportion of each subpopulation sampled and inflammatory markers. Another possible explanation is that the long-term patient transmitted *Campylobacter* to other human patients.


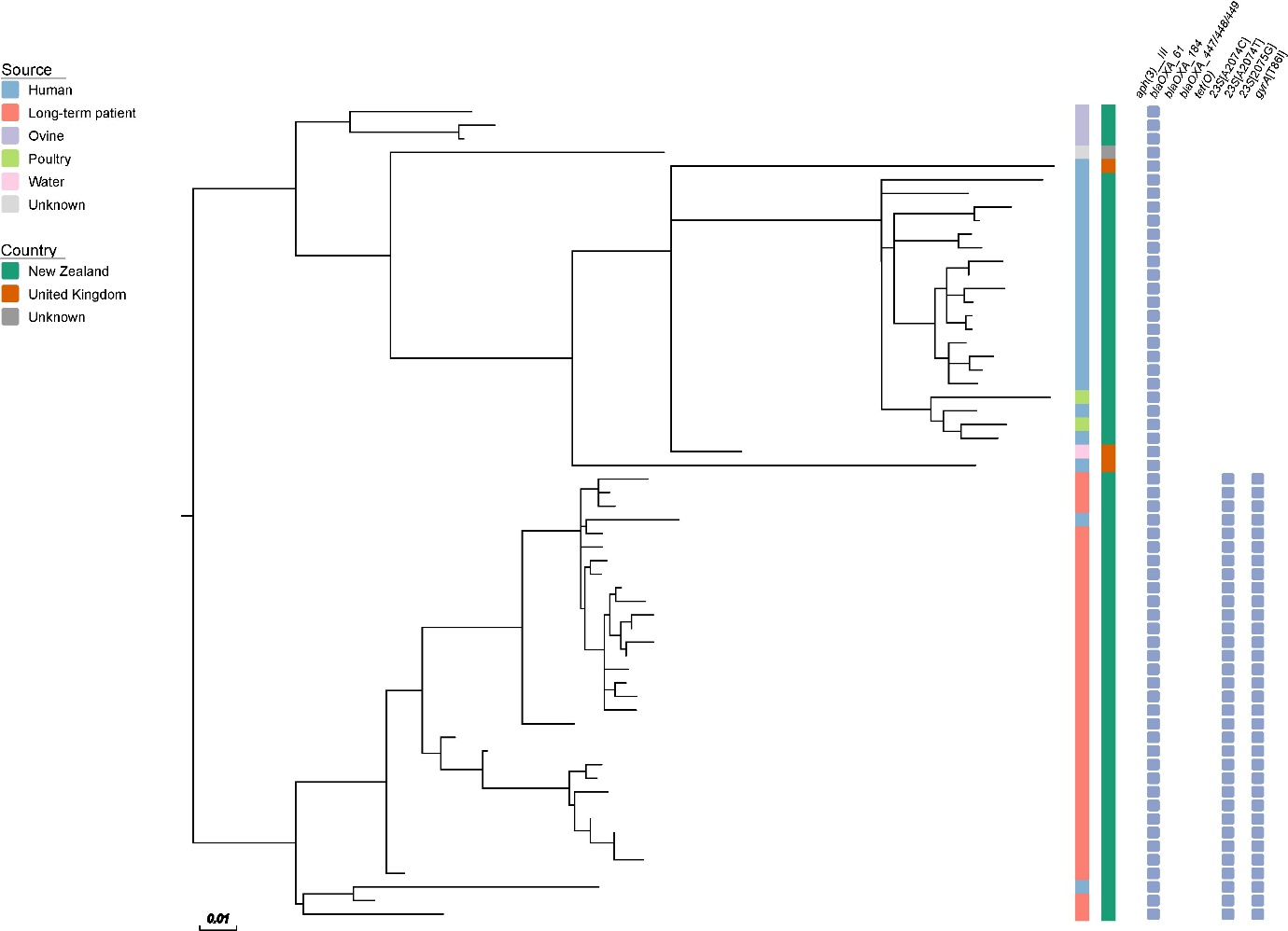


**Figure S5.** Maximum likelihood tree of clade 18 of the ST45 isolates. Colored bars represent isolate metadata and the presence-absence matrix represents AMR genes.

**References**

1. Bloomfield SJ, Midwinter AC, Biggs PJ, French NP, Marshall JC, Hayman DTS, et al. Long-term colonization by *Campylobacter jejuni* within a human host: Evolution, antimicrobial resistance, and adaptation. J Infect Dis. 2018;217:103–11.
