# Supplementary material for: Genomic adaptations of Campylobacter jejuni to long-term human colonization
Source: Gut Pathog. 2021 Dec 10;13:72. doi: 10.1186/s13099-021-00469-7 (PMC8665580; doi:10.1186/s13099-021-00469-7)
Supplement: Supplementary file 4 — Additional file 4. New Zealand patient phylogenetic analysis. [file 13099_2021_469_MOESM4_ESM.docx]

**New Zealand patient phylogenetic analysis**

**New Zealand patient clock signal detection**

To determine if there was a clock signal from the isolates collected from the New Zealand patient, IQ-TREE [1] was used to form a maximum likelihood tree from the identified non-recombinant SNPs and TempEst [2] was used to was used to test for temporal signal (Figure S6): the root-to-tip divergence and date of collection were highly correlated (R^2^ = 0.92).


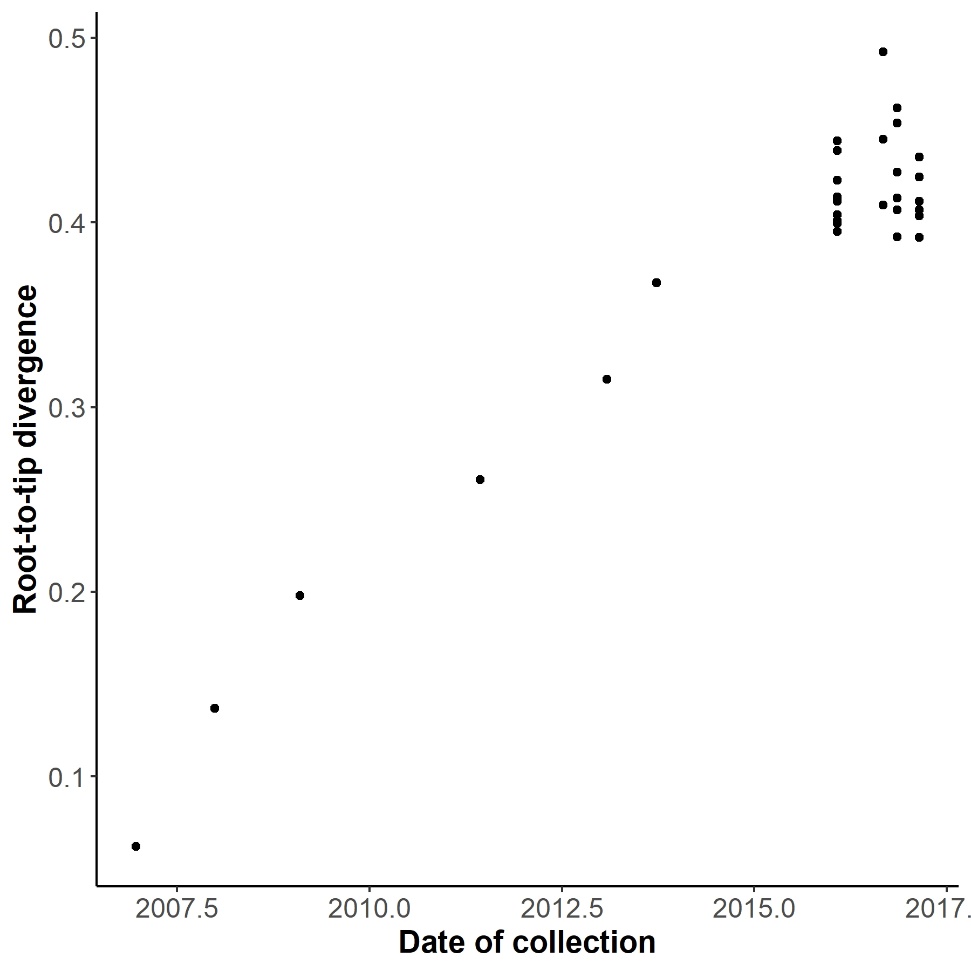


**Figure S6.** TempEst output for 31 ST45 isolates collected from the New Zealand patient.

**New Zealand patient substitution modelling**

bModelTest [3] was used to choose the substitution model for the 31 *C. jejuni* ST45 isolates collected from the New Zealand patient, based on 248 non-recombinant SNPs, and 584,548 adenine, 254,724 cytosine, 253,882 guanine and 576,175 thymine nucleotides constant sites for 50 million steps. No model was widely supported so a standard 123456 Generalise Time Reversible (GTR) model was used [4].

**New Zealand patient model comparison**

The 31 ST45 isolates phylogenetics were modelled by placing the 248 SNPs shared by these isolates into BEAST 2.5 [5], and running combinations of molecular clock (strict, random [6] and uncorrelated relaxed [7]) and tree (constant coalescent and Extended Bayesian Skyline [8]) models for 100 million steps. Model combinations with sufficient chain convergence (posterior estimated sample size (ESS) over 200) were compared using Nested Sampling (NS) [9] with 10,000 chains, 5,000 subchains and 10 particles (Table S2). The model combination with the highest Marginal Likelihood Estimate (MLE) was further analysed.

**Table S2**. ESS and MLE values of model combinations in BEAST

| Clock | Tree | ESS  (posterior) | NS  (MLE) | Standard deviation |
| --- | --- | --- | --- | --- |
| Random | Constant | 24 |  |  |
| Random | EBS | 13 |  |  |
| Relaxed | Constant | 1887 | -2187735.9 | 3.02 |
| Relaxed | EBS | 2184 | -2187738.5 | 3.16 |
| Strict | Constant | 3540 | -2187743.6 | 3.18 |
| **Strict** | **EBS** | **3041** | **-2187731.5** | **3.10** |

Chosen model combination is boldened.

The Extended Bayesian Skyline model estimated that the effective population size for *Campylobacter* from the New Zealand patient was consistent from 2004-2015, where it then increased (Figure S7). This increase coincides with the onset of collecting multiple isolates from fecal samples [10] and with more frequent sample collections. Previous studies have demonstrated that different sampling strategies can affect effective population size estimates of skyline plots [11,12]. Therefore, we cannot rule out that the change in effective population size observed in this study is not due to a change in sampling strategy.


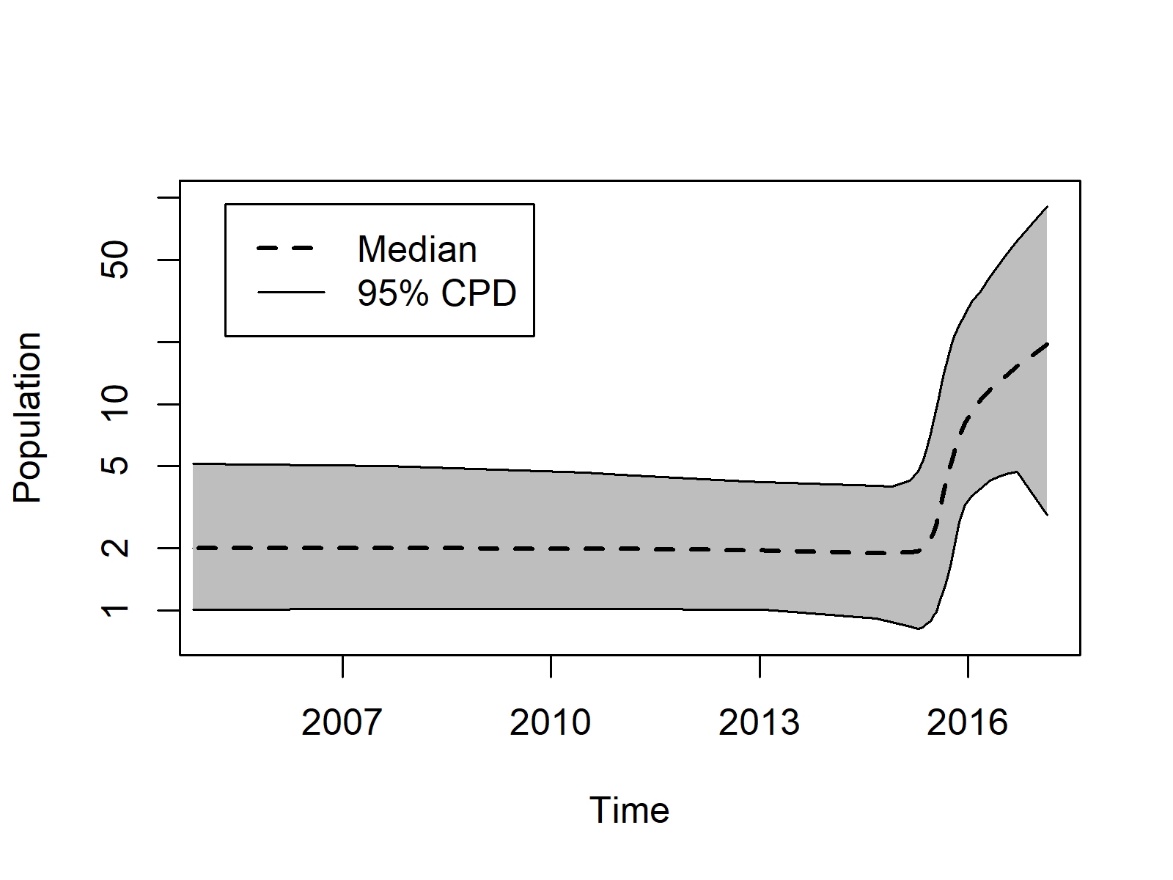


**Figure S7.** Extended Bayesian Skyline plot of the change in effective population size of the 31 ST45 isolates collected from the New Zealand patient.

Previously, we concluded that the New Zealand patient had been continuously colonized with *Campylobacter* beginning sometime between 1998 and 2006, as this was when the 16 isolates previously sequenced from the patient were estimated to share a date of common ancestor [10]. This is consistent with when the patient started suffering from daily episodes of diarrhea in 2000. In this study we were able to provide a narrower range of date of common ancestors for the 31 isolates between 2002 and 2005. This increased precision can be attributed to the larger number of isolates analyzed, the removal of recombination and the selection of optimal methods for ancestral state reconstruction by trialing a range of substitution, clock and tree models. Interestingly, this more precise estimate is after the time the patient started suffering from episodes of diarrhea. There are three possible explanations for this. First, the initial diarrhea episodes were not due to *Campylobacter*, but the cause of the diarrhea allowed the patient to be colonized with *Campylobacter*. Second, the patient was colonized with *Campylobacter* prior to the estimated date of common ancestor, but the population descending from the initial infection went through genetic changes that erased lineages that coalesced from the samples and sequenced isolates to the initial infecting strains. Third, the patient was colonized by multiple, unrelated *Campylobacter* strains, only one of which persisted and diversified. The second and third explanations rely on selective sweeps in the host, for which there is evidence for in the selection of AMR mutants following the prescription of antimicrobial agents. Earlier fecal samples collected at the time the patient started suffering from diarrhea would be required to determine which explanation is responsible, but such samples do not exist.

**References**

1. Nguyen L-T, Schmidt HA, von Haeseler A, Minh BQ. IQ-TREE: a fast and effective stochastic algorithm for estimating maximum-likelihood phylogenies. Mol Biol Evol. 2015;32:268–74.

2. Rambaut A, Lam TT, Carvalho LM, Pybus OG. Exploring the temporal structure of heterochronous sequences using TempEst (formerly Path-O-Gen). Virus Evol. 2016;2:1–7.

3. Bouckaert RR, Drummond AJ. bModelTest: Bayesian phylogenetic site model averaging and model comparison. BMC Evol Biol. 2017;17:1–11.

4. Tavare S. Some probabilistic and statistical problems in the analysis of DNA sequences. Am Math Soc. 1986;17:57–86.

5. Bouckaert R, Heled J, Kühnert D, Vaughan T, Wu C-H, Xie D, et al. BEAST 2: A software platform for Bayesian evolutionary analysis. PLoS Comput Biol. 2014;10:1–6.

6. Drummond AJ, Suchard MA. Bayesian random local clocks, or one rate to rule them all. BMC Biol. 2010;8:1–12.

7. Drummond AJ, Ho SYW, Phillips MJ, Rambaut A. Relaxed phylogenetics and dating with confidence. PLoS Biol. 2006;4:699–710.

8. Heled J, Drummond AJ. Bayesian inference of population size history from multiple loci. BMC Evol Biol. 2008;8:1–15.

9. Russel PM, Brewer BJ, Klaere S, Bouckaert RR. Model selection and parameter inference in phylogenetics using nested sampling. Syst Biol. 2018;68:219–33.

10. Bloomfield SJ, Midwinter AC, Biggs PJ, French NP, Marshall JC, Hayman DTS, et al. Long-term colonization by *Campylobacter jejuni* within a human host: Evolution, antimicrobial resistance, and adaptation. J Infect Dis. 2018;217:103–11.

11. Hall MD, Woolhouse MEJ, Rambaut A. The effects of sampling strategy on the quality of reconstruction of viral population dynamics using Bayesian skyline family coalescent methods: A simulation study. Virus Evol. 2016;2:1–14.

12. Heller R, Chikhi L, Siegismund HR. The confounding effect of population structure on Bayesian skyline plot inferences of demographic history. PLoS One. 2013;8:1–10.
