# Supplementary material for: Genomic adaptations of Campylobacter jejuni to long-term human colonization
Source: Gut Pathog. 2021 Dec 10;13:72. doi: 10.1186/s13099-021-00469-7 (PMC8665580; doi:10.1186/s13099-021-00469-7)
Supplement: Supplementary file 5 — Additional file 5. New Zealand patient clade gene analysis. [file 13099_2021_469_MOESM5_ESM.docx]

**New Zealand patient clade gene analysis**

Isolates collected from the New Zealand patient revealed that two clades had evolved. Four frameshifts and eleven non-synonymous SNPs were found that differed between the two clades (Figure S8). A disproportionate number of the genes affected were involved in signal transduction (COG functional group T), but this only represented three genes from this functional group (Figure S9).


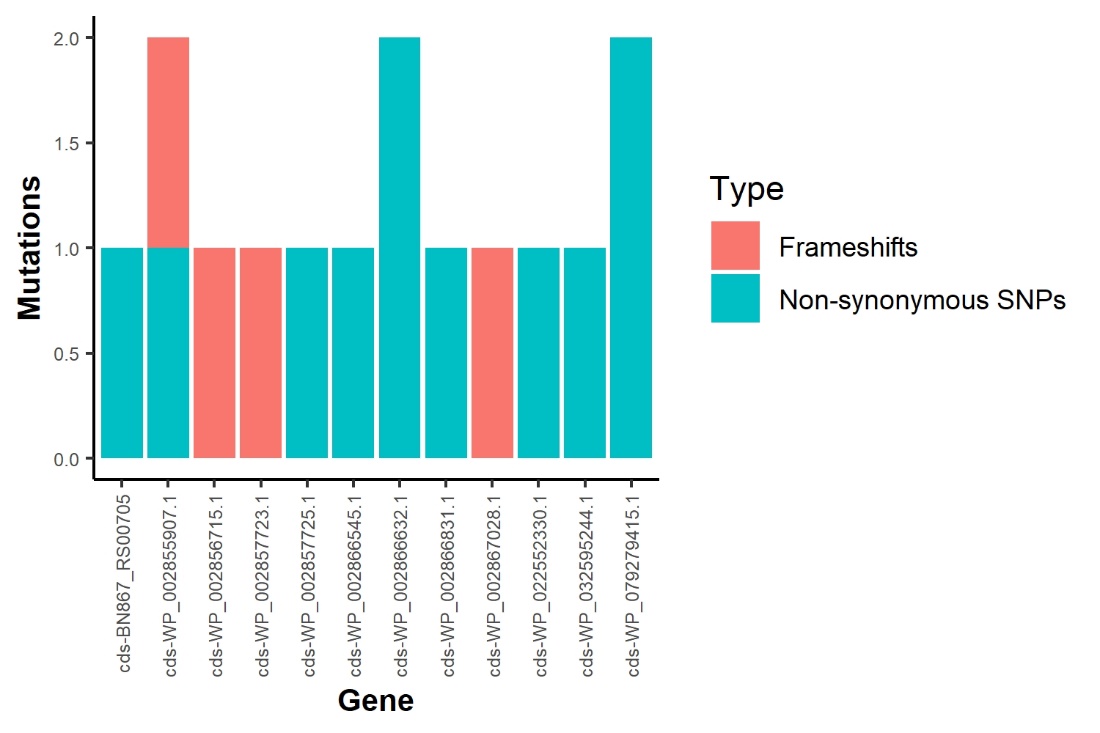


**Figure S8**. Bar plot of the number of frameshifts (red) and non-synonymous SNPs (blue) that differed between the two clades identified amongst isolates collected from the New Zealand patient.


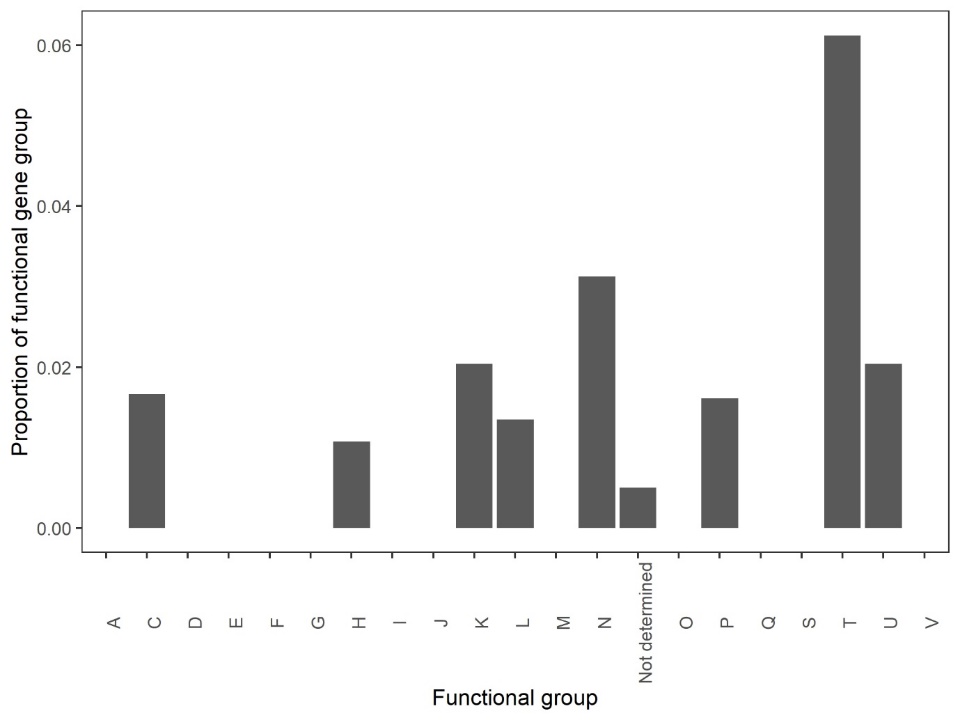


**Figure S9**. Bar plot of the proportion of genes belonging to each functional group that contained frameshifts or non-synonymous SNPs between the two clades identified amongst the isolates collected from the New Zealand patient.
