# Supplementary material for: Genomic adaptations of Campylobacter jejuni to long-term human colonization
Source: Gut Pathog. 2021 Dec 10;13:72. doi: 10.1186/s13099-021-00469-7 (PMC8665580; doi:10.1186/s13099-021-00469-7)
Supplement: Supplementary file 6 — Additional file 6. New Zealand patient gene number modeling. [file 13099_2021_469_MOESM6_ESM.docx]

**New Zealand patient gene number modelling**

The total number of genes and pseudogenes from each isolate collected from the New Zealand patient were modelled using linear regression models with date of collection and clade as explanatory variables (Figures S10-S12). Multiple isolates from two clades were collected from the last four samples, so the mean number of genes and pseudogenes for each clade at each sample were calculated (Figure S13).


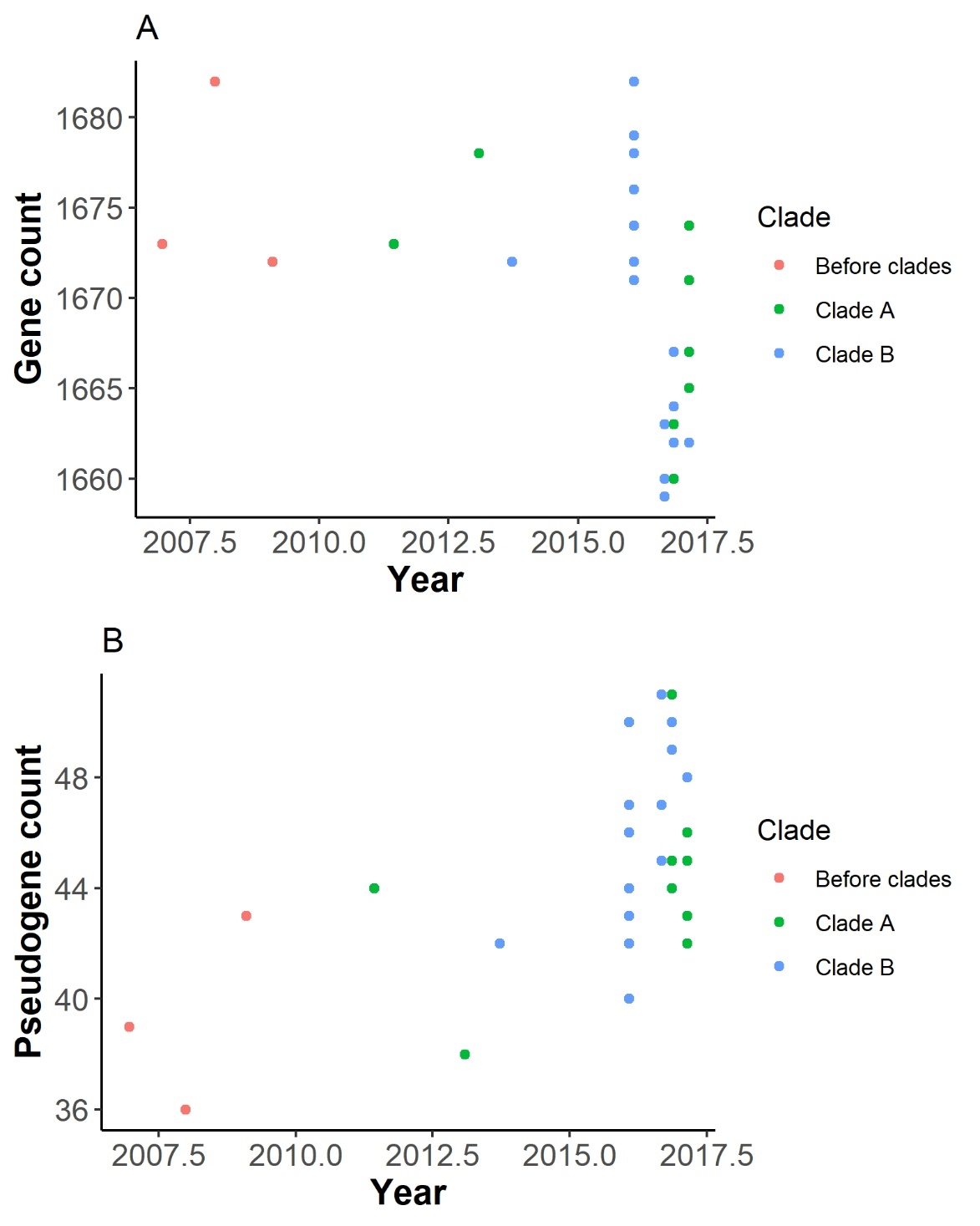


**Figure S10.** Scatterplots of the number of genes (A) and pseudogenes (B) contained by 31 ST45 isolates collected from the New Zealand patient versus date of collection and colored by clade.


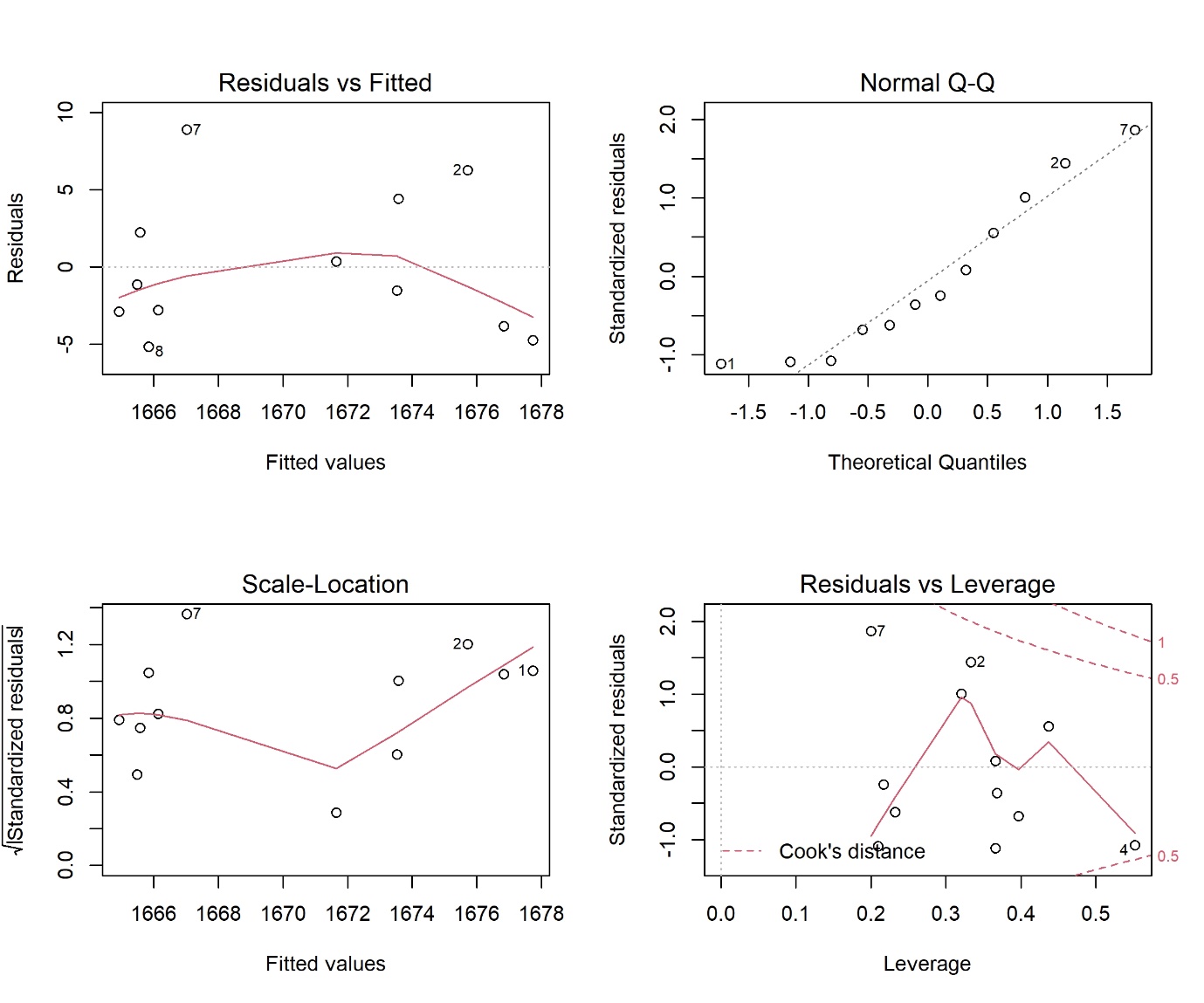


**Figure S11**. Regression plots of the linear regression model used to model the number of genes from the New Zealand patient.


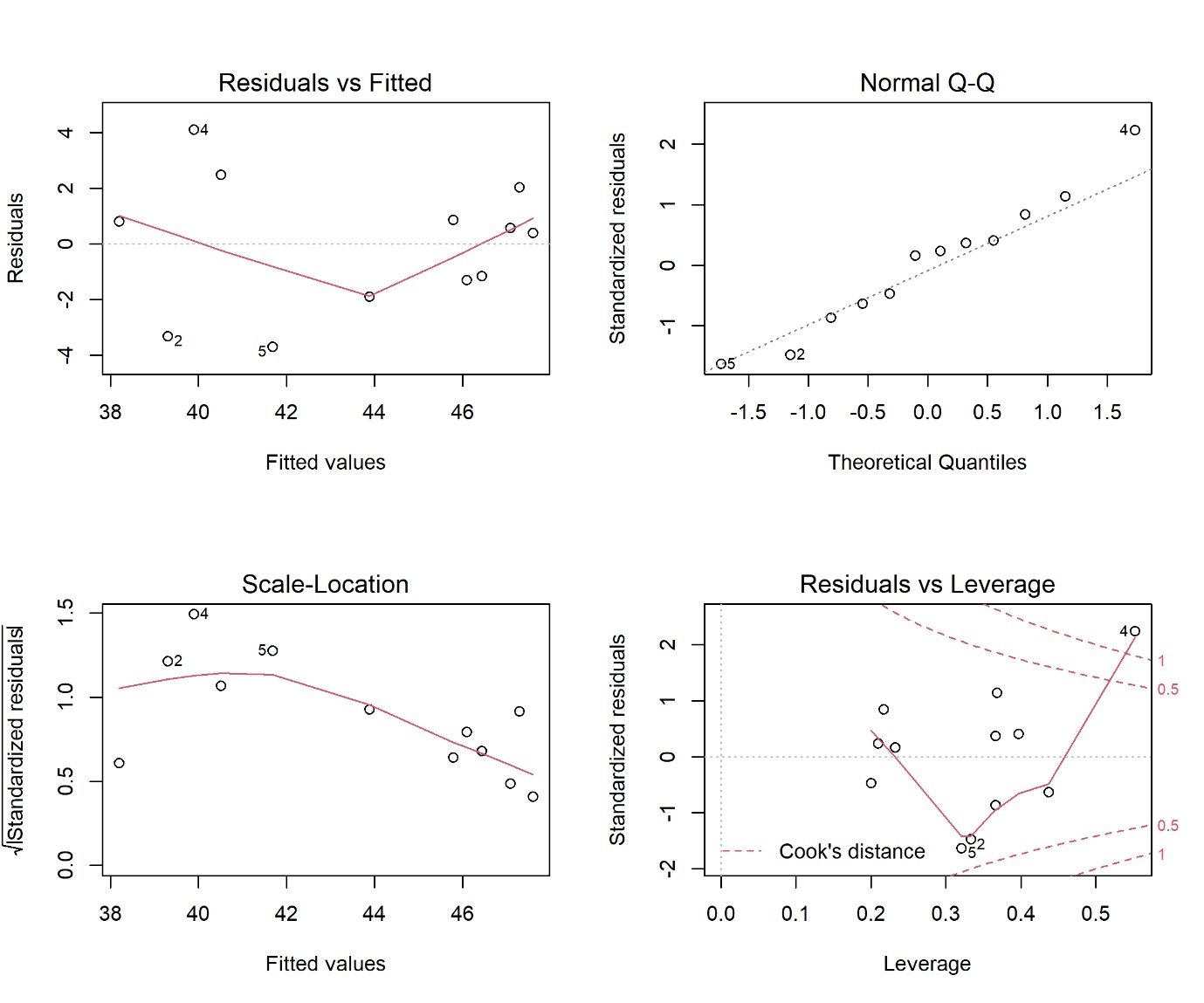


**Figure S12**. Regression plots of the linear regression model used to model the number of pseudogenes from the New Zealand patient.


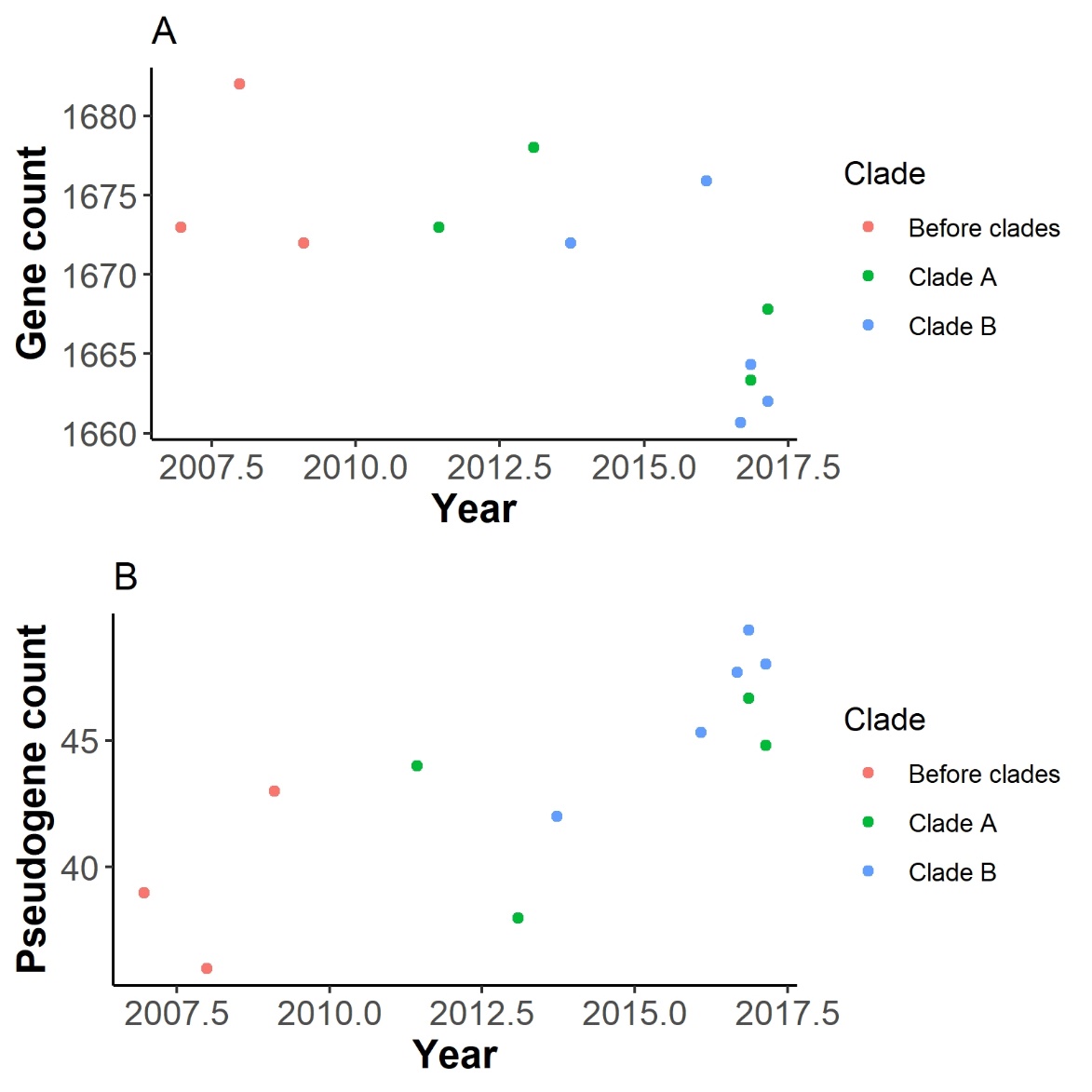


**Figure S13.** Scatterplots of the number of genes (A) and pseudogenes (B) contained by 31 ST45 isolates collected from the New Zealand patient versus date of collection and colored by clade that were modelled.

Date of collection was not associated with the number of genes (p= 0.0635) or the number of pseudogenes (p=0.0501). Partial-F tests found that clade was not associated with the number of genes (p=0.5664) or pseudogenes (p=0.5386). The models explained some of the variation in the variance of the number of genes (R^2^=0.3761) or pseudogenes (R^2^=0.5774).

**New Zealand patient gene number linear regression model**

| Linear regression formula: |
| --- |
| Genes ~ Date + Factor(Clade) |

Residuals:

| Minimum | First quartile | Median | Third quartile | Maximum |
| --- | --- | --- | --- | --- |
| -5.172 | -3.146 | -1.340 | 2.771 | 8.886 |

| Coefficient | Estimate | Standard error | t-value | p-value |
| --- | --- | --- | --- | --- |
| Intercept | 5638 | 1841 | 3.063 | 0.0155 |
| Date | -1.973 | 0.9168 | -2.152 | 0.0635 |
| Clade A | 7.916 | 7.301 | 1.084 | 0.3098 |
| Clade B | 7.255 | 8.365 | 0.867 | 0.4111 |

| Statistic | Value |
| --- | --- |
| Residual standard error | 5.325 on 8 degrees of freedom |
| Multiple R-squared | 0.5462 |
| Adjusted R-squared | 0.3761 |
| F-statistic | 3.21 on 3 and 8 degrees of freedom |
| p-value | 0.08313 |

| Partial-F test genes and clades | | |  |  |  |  |
| --- | --- | --- | --- | --- | --- | --- |
| Model | **Residual degrees of freedom** | **Residual sum of squares** | **Degrees of freedom** | **Sum of squares** | **F-value** | **p-value** |
| Gene | 8 | 226.9 |  |  |  |  |
| Gene minus clade | 10 | 261.5 | -2 | -34.64 | 0.6108 | 0.5664 |

**New Zealand patient pseudogene number linear regression model**

| Linear regression formula: |
| --- |
| Pseudogenes ~ Date + Factor(Clade) |

Residuals:

| Minimum | First quartile | Median | Third quartile | Maximum |
| --- | --- | --- | --- | --- |
| -3.685 | -1.446 | 0.4898 | 1.169 | 4.107 |

| Coefficient | Estimate | Standard error | t-value | p-value |
| --- | --- | --- | --- | --- |
| Intercept | -2146 | 948.3 | -2.263 | 0.0535 |
| Date | 1.088 | 0.4722 | 2.304 | 0.0501 |
| Clade-A | -3.162 | 3.760 | -0.841 | 0.4249 |
| Clade-B | -1.663 | 4.309 | -0.386 | 0.7095 |

| Statistic | Value |
| --- | --- |
| Residual standard error | 2.743 on 8 degrees of freedom |
| Multiple R-squared | 0.6923 |
| Adjusted R-squared | 0.5774 |
| F-statistic | 6.01 on 3 and 8 degrees of freedom |
| p-value | 0.01906 |

**Partial-F test pseudogenes and clades**

| Model | Residual degrees of freedom | Residual sum of squares | Degrees of freedom | Sum of squares | F-value | p-value |
| --- | --- | --- | --- | --- | --- | --- |
| Pseudogenes | 8 | 60.19 |  |  |  |  |
| Pseudogenes minus clade | 10 | 70.26 | -2 | -10.07 | 0.6693 | 0.5386 |
