# Supplementary material for: Genomic adaptations of Campylobacter jejuni to long-term human colonization
Source: Gut Pathog. 2021 Dec 10;13:72. doi: 10.1186/s13099-021-00469-7 (PMC8665580; doi:10.1186/s13099-021-00469-7)
Supplement: Supplementary file 7 — Additional file 7. United Kingdom patient phylogenetic analysis. [file 13099_2021_469_MOESM7_ESM.docx]

**United Kingdom phylogenetic analysis**

**United Kingdom patient substitution modelling**

bModelTest [1] was used to choose the substitution model for the 22 *C. jejuni* ST45 isolates collected from the United Kingdom patient, based on 122 non-recombinant SNPs and using the same methodology appied to the New Zealand patient isolates. No model was widely supported so a standard 123456 Generalise Time Reversible (GTR) model was used [2].

**United Kingdom patient model comparison**

The 22 ST45 isolates phylogenetics were modelled by placing the 122 SNPs shared by these isolates into BEAST 2.5 [3] and using the same methodology appied to the New Zealand patient isolates (Figure S14).

**Table S3**. ESS and MLE values of United Kingdom patient model combinations in BEAST

| Clock | Tree | ESS  (posterior) | NS  (MLE) | Standard deviation |
| --- | --- | --- | --- | --- |
| Random | Constant | 11 |  |  |
| Random | EBS | 4 |  |  |
| Relaxed | Constant | 3308 | -2185399.5 | 2.47 |
| Relaxed | EBS | 4 |  |  |
| **Strict** | **Constant** | **3814** | **-2185395.6** | **2.22** |
| Strict | EBS | 4 |  |  |

Chosen model combination is boldened.


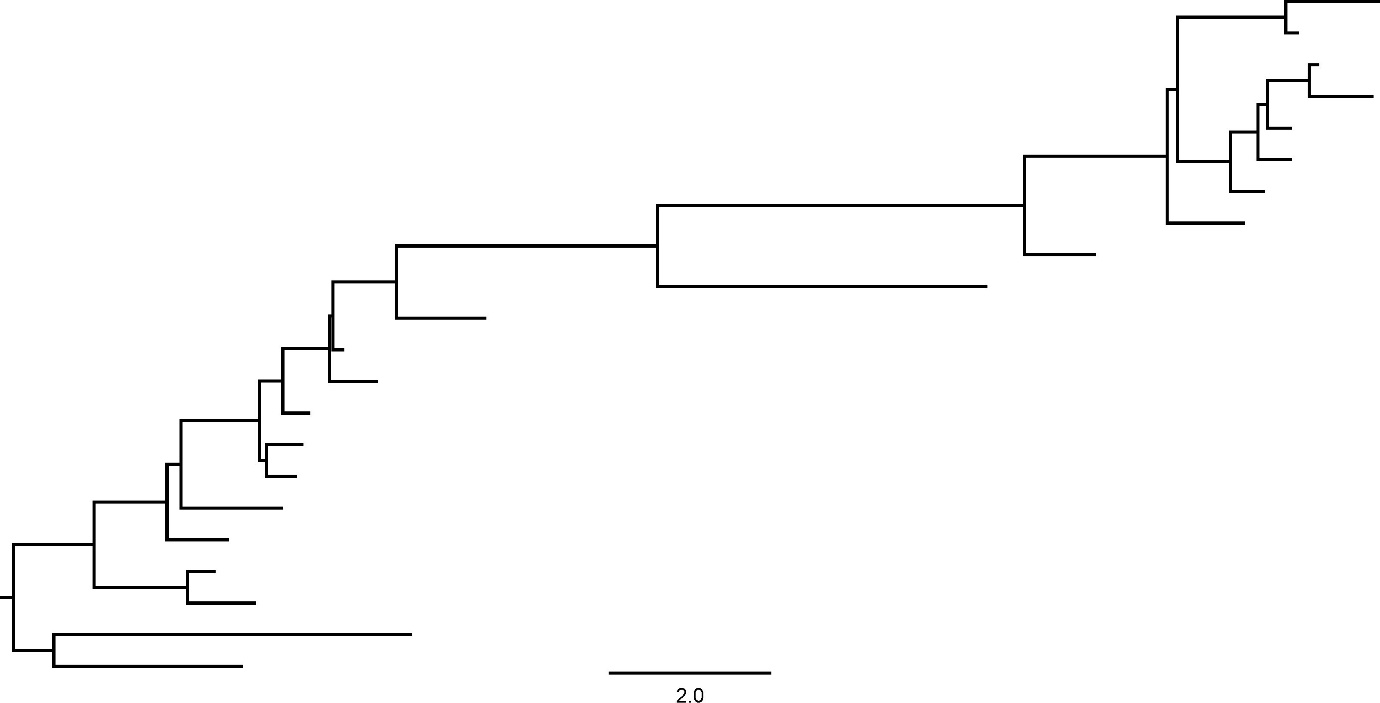


**Figure S14.** Maximum credibility tree of 22 ST45 isolates collected from the United Kingdom patient using 122 non-recombinant SNPs. Scale represents the length of two years.

**References**

1. Bouckaert RR, Drummond AJ. bModelTest: Bayesian phylogenetic site model averaging and model comparison. BMC Evol Biol. 2017;17:1–11.

2. Tavare S. Some probabilistic and statistical problems in the analysis of DNA sequences. Am Math Soc. 1986;17:57–86.

3. Bouckaert R, Heled J, Kühnert D, Vaughan T, Wu C-H, Xie D, et al. BEAST 2: A software platform for Bayesian evolutionary analysis. PLoS Comput Biol. 2014;10:1–6.
