# Supplementary material for: Genomic adaptations of Campylobacter jejuni to long-term human colonization
Source: Gut Pathog. 2021 Dec 10;13:72. doi: 10.1186/s13099-021-00469-7 (PMC8665580; doi:10.1186/s13099-021-00469-7)
Supplement: Supplementary file 8 — Additional file 8. United Kingdom patient gene number modeling. [file 13099_2021_469_MOESM8_ESM.docx]

**United Kingdom patient gene number modeling**

The total number of genes and pseudogenes from each isolate collected from the United Kingdom patient were modelled using linear regression models with date of collection as the explanatory variable (Figures S15-S17). Date of collection was found to be negatively associated with the number of genes (p= 0.01290) and positively associated the number of pseudogenes (p=0.00175). The models explained some of the variance in the number of genes (R^2^=0.4246) or pseudogenes (R^2^=0.6053).


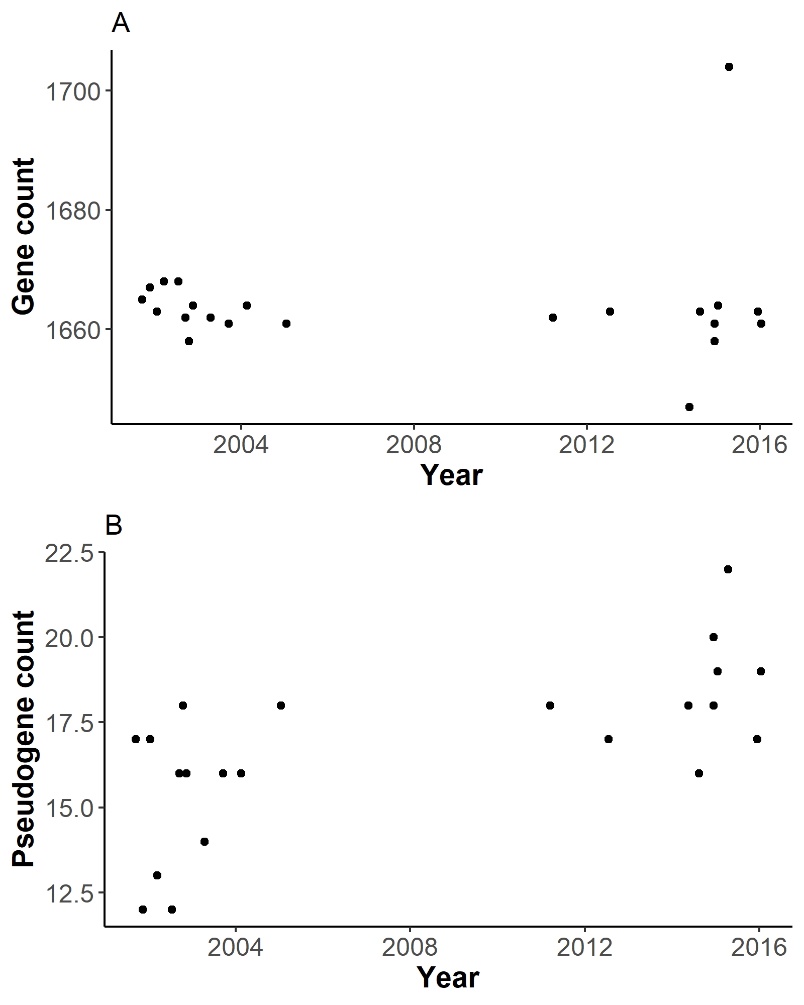


**Figure S15.** Scatterplots of the number of genes (A) and pseudogenes (B) contained by 22 ST45 isolates collected from the United Kingdom patient versus date of collection.


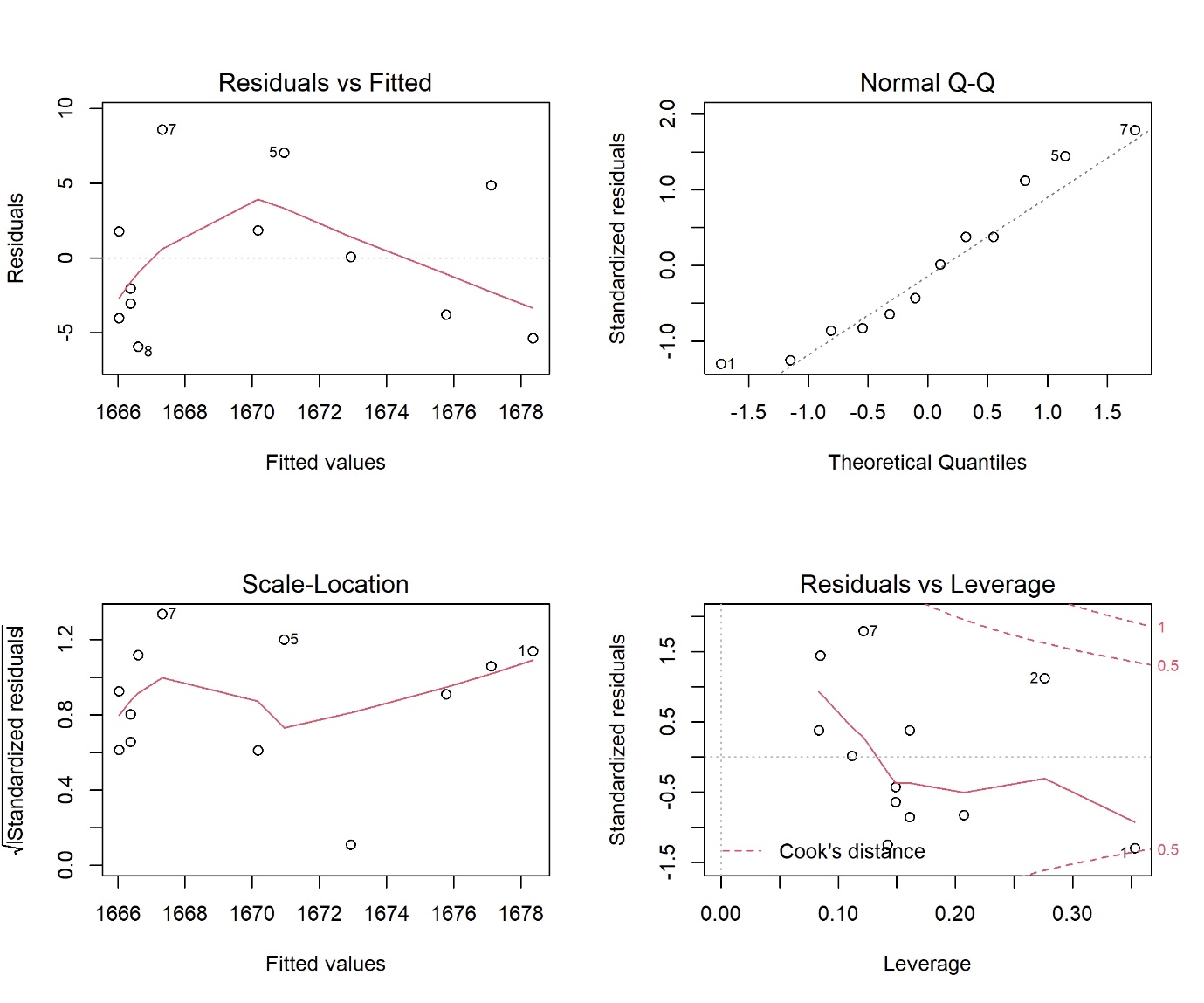


**Figure S16**. Regression plots of the linear regression model used to model the number of genes from the United Kingdom patient.


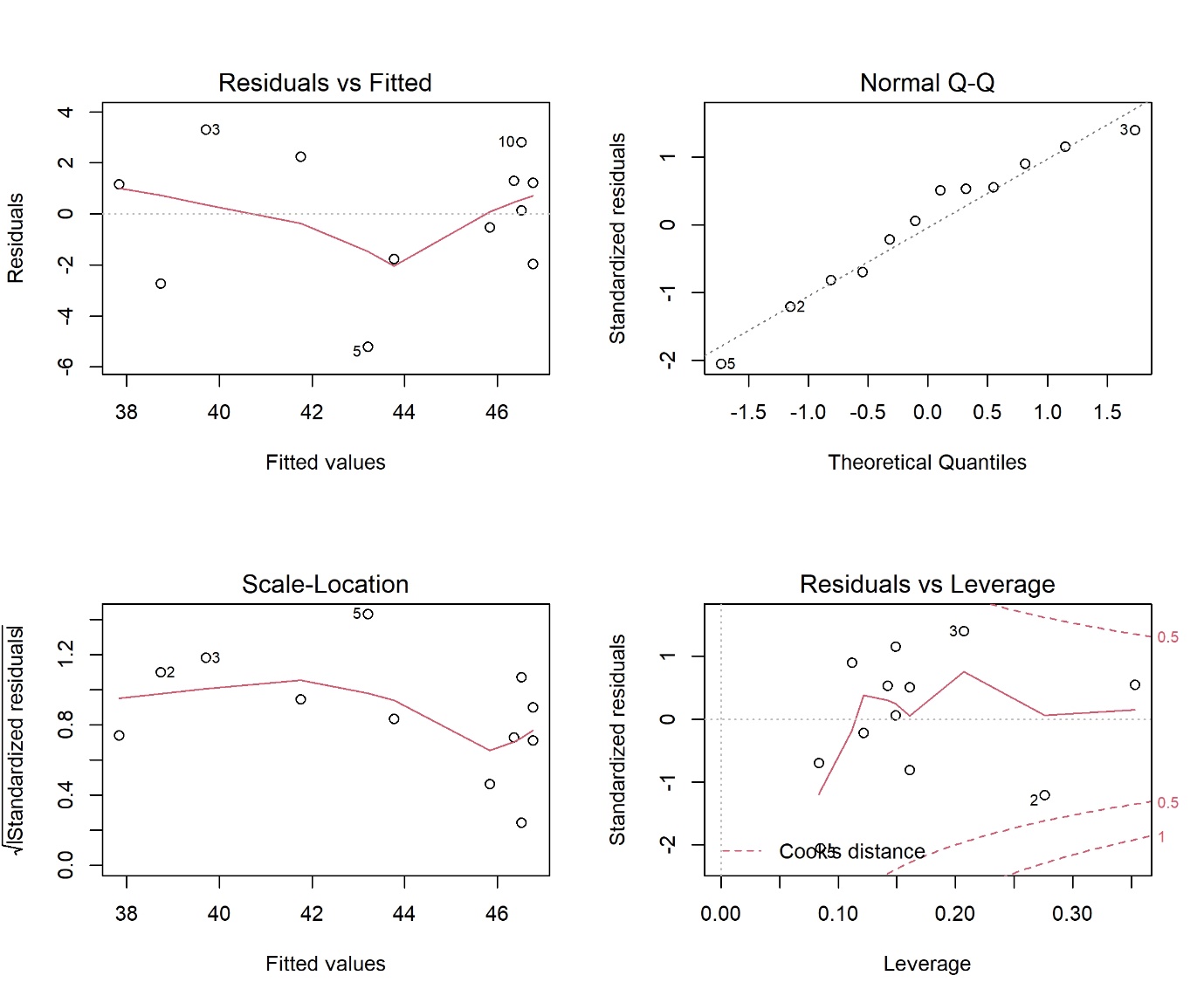


**Figure S17**. Regression plots of the linear regression model used to model the number of pseudogenes from the United Kingdom patient.

**United Kingdom patient gene number linear regression model**

| Linear regression formula: |
| --- |
| Genes ~ Date |

Residuals:

| Minimum | First quartile | Median | Third quartile | Maximum |
| --- | --- | --- | --- | --- |
| -5.932 | -3.838 | -0.9917 | 2.596 | 8.580 |

| Coefficient | Estimate | Standard error | t-value | p-value |
| --- | --- | --- | --- | --- |
| Intercept | 4109 | 807.5 | 5.088 | 4.72 x 10^-4^ |
| Date | -1.211 | 0.401 | -3.020 | 0.01290 |

| Statistic | Value |
| --- | --- |
| Residual standard error | 5.114 on 10 degrees of freedom |
| Multiple R-squared | 0.4769 |
| Adjusted R-squared | 0.4246 |
| F-statistic | 9.118 on 1 and 10 degrees of freedom |
| p-value | 0.0129 |

**United Kingdom patient pseudogene number linear regression model**

| Linear regression formula: |
| --- |
| Pseudogenes ~ Date |

Residuals:

| Minimum | First quartile | Median | Third quartile | Maximum |
| --- | --- | --- | --- | --- |
| -5.205 | -1.820 | 0.6588 | 1.541 | 3.298 |

| Coefficient | Estimate | Standard error | t-value | p-value |
| --- | --- | --- | --- | --- |
| Intercept | -1726 | 418.5 | -4.123 | 0.00207 |
| Date | 0.8787 | 0.2079 | 4.228 | 0.00175 |

| Statistic | Value |
| --- | --- |
| Residual standard error | 2.651 on 10 degrees of freedom |
| Multiple R-squared | 0.6412 |
| Adjusted R-squared | 0.6053 |
| F-statistic | 17.87 on 1 and 10 degrees of freedom |
| p-value | 0.001751 |
