# Supplementary material for: Genomic adaptations of Campylobacter jejuni to long-term human colonization
Source: Gut Pathog. 2021 Dec 10;13:72. doi: 10.1186/s13099-021-00469-7 (PMC8665580; doi:10.1186/s13099-021-00469-7)
Supplement: Supplementary file 10 — Additional file 10. Long-term patients’ pangenome function analysis. [file 13099_2021_469_MOESM10_ESM.docx]

**Long-term patients’** **pangenome function analysis**

Isolates from the New Zealand patient had a pangenome of 1,722 genes with an accessory genome of 86 genes, whilst the isolates collected from the United Kingdom had a pangenome of 1,743 genes and an accessory genome of 103 genes. The accessory genome for each patient was not associated with any functional group (Figure S18). The exception was RNA processing and modification (COG group A) in the New Zealand patient, but this is because only two of these genes were identified and one was accessory. In both patients, genes that were stably-lost were identified, but these were not associated with any specific functional group either.


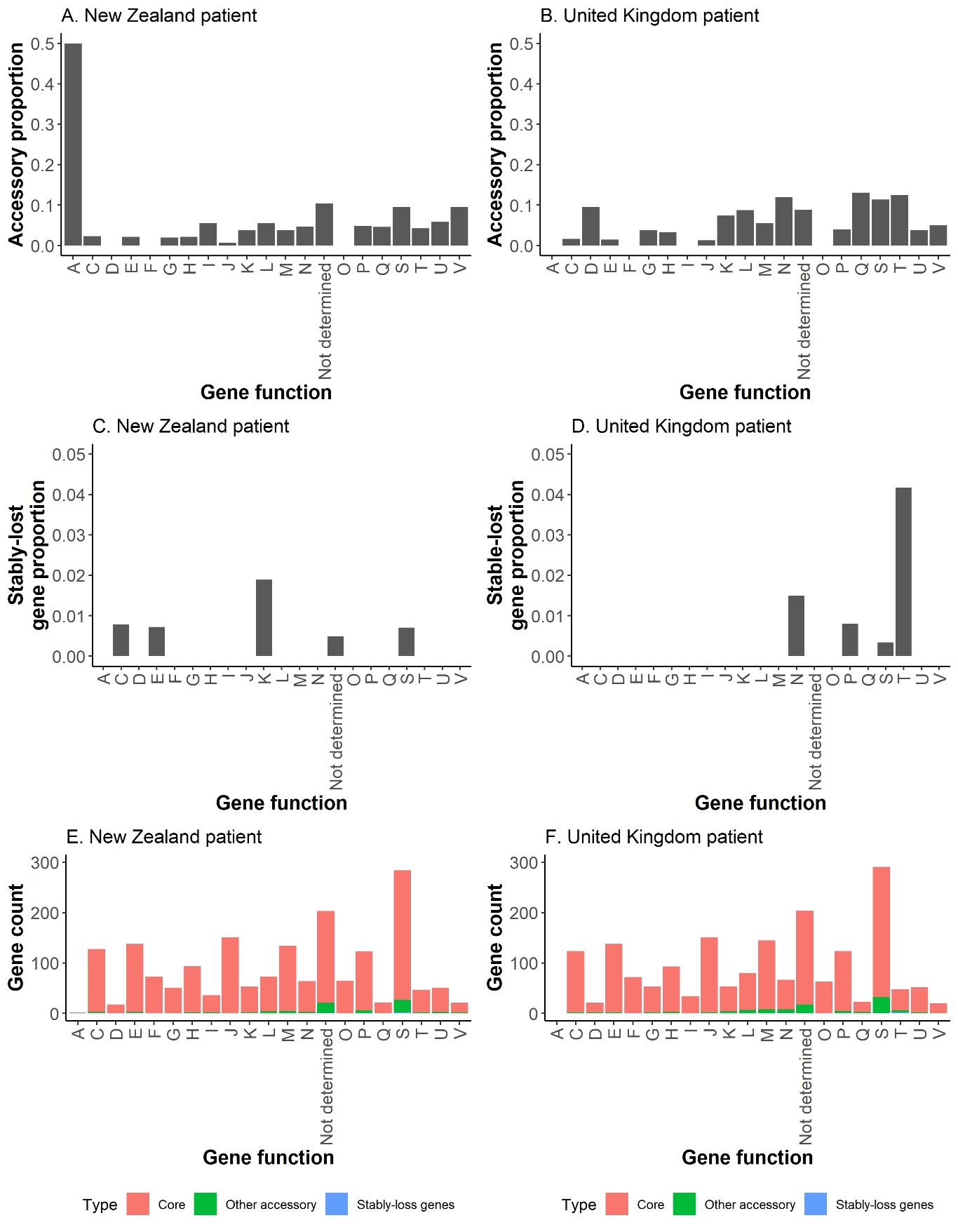


**Figure S18**. Bar plots of the proportion of genes belonging to each functional group that were accessory in the (A and B) and stably-lost (C and D), and the total number of genes in these functional groups that were stably-lost, other accessory or core (E and F) in New Zealand (A, C and E) and United Kingdom (B, D and F) patients.
