# Supplementary material for: Genomic adaptations of Campylobacter jejuni to long-term human colonization
Source: Gut Pathog. 2021 Dec 10;13:72. doi: 10.1186/s13099-021-00469-7 (PMC8665580; doi:10.1186/s13099-021-00469-7)
Supplement: Supplementary file 11 — Additional file 11. Long-term patients’ gene variation function analysis. [file 13099_2021_469_MOESM11_ESM.docx]

**Long-term patients’** **gene variation function analysis**

The isolates collected from the New Zealand patient shared 134 core non-synonymous SNPs and 23 core frameshifts in 92 genes, whilst the isolates collected from the United Kingdom patient shared 88 core non-synonymous SNPs and 31 core frameshifts in 81 genes (Figure S19). Of these, 23 non-synonymous SNPs and one frameshifts from the New Zealand patient isolates, and 15 non-synonymous SNPs and three frameshifts from the United Kingdom patient isolates were stably-inherited in 32 genes. One of the stably-inherited frameshifts from the New Zealand patient isolates and three from the United Kingdom patient isolates were not found in genes.


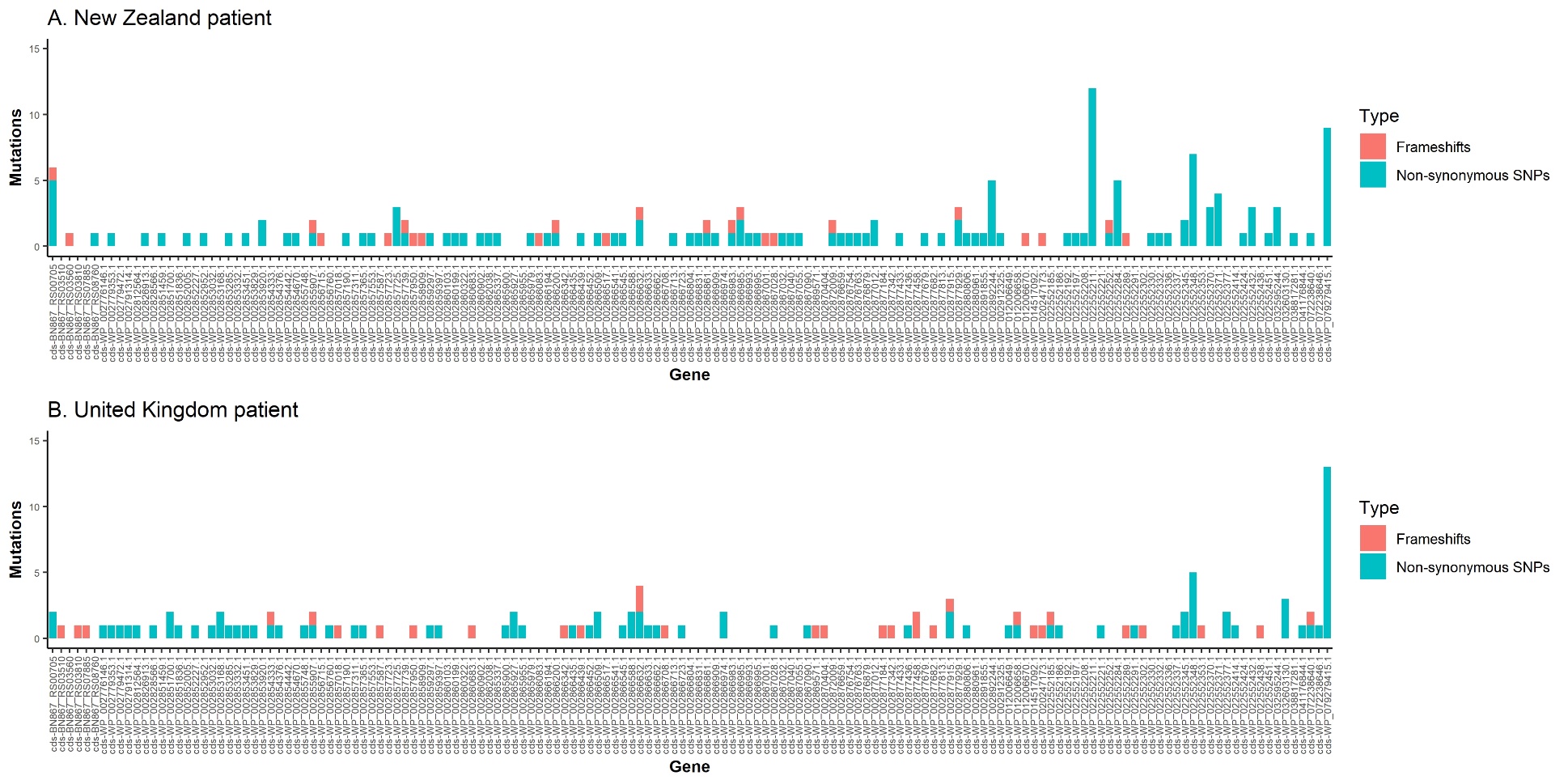


**Figure S19**. Bar plots of the number of frameshifts (red) and non-synonymous SNPs (blue) in genes from isolates collected from the New Zealand (A) and United Kingdom (B) patients.


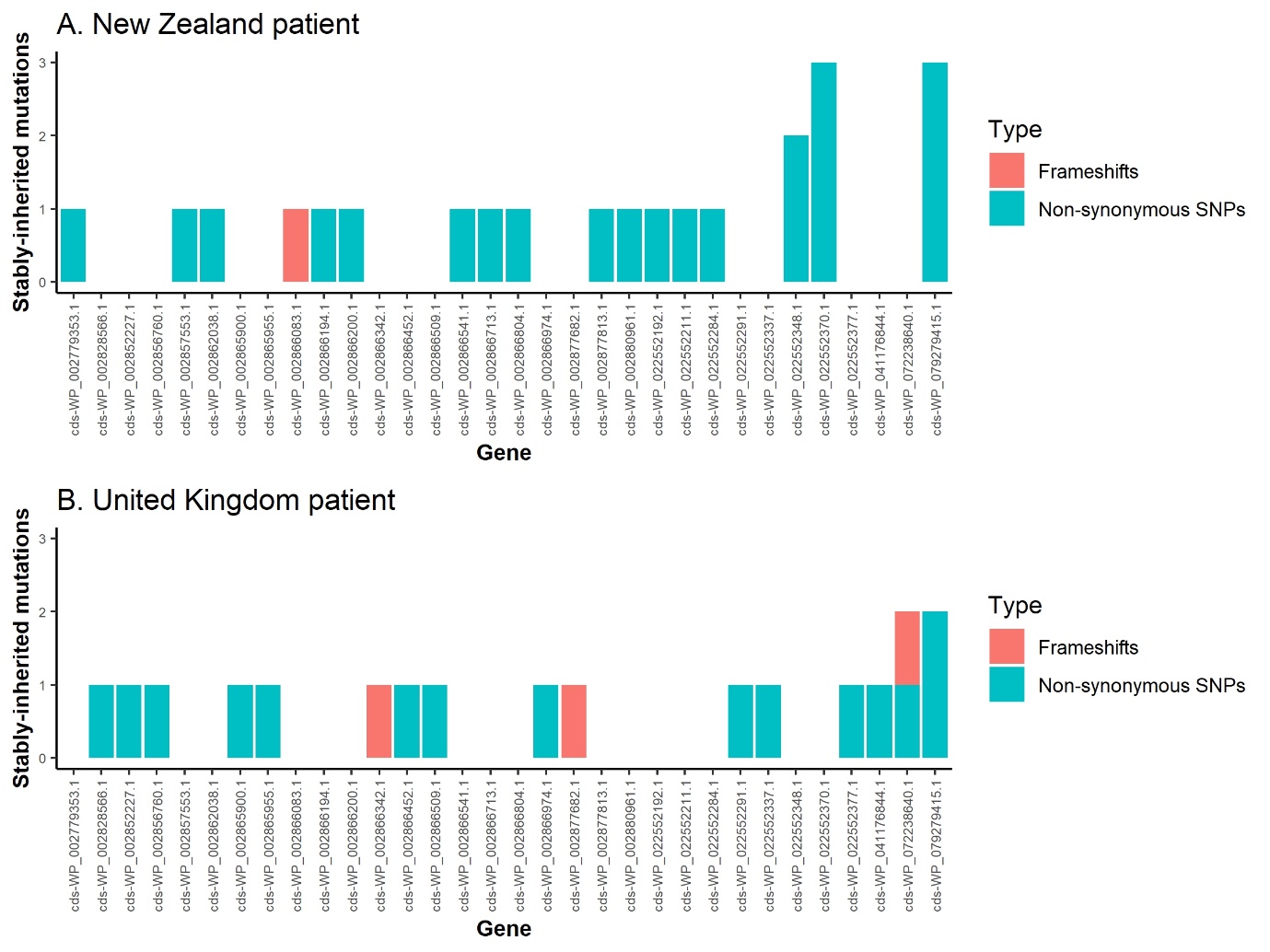


**Figure S20**. Bar plots of the number of stably-inherited frameshifts (red) and non-synonymous SNPs (blue) in genes from isolates collected from the New Zealand (A) and United Kingdom (B) patients.
